# Germline *BRCA2* mutations drive prostate cancers with distinct evolutionary trajectories

**DOI:** 10.1038/ncomms13671

**Published:** 2017-01-09

**Authors:** Renea A. Taylor, Michael Fraser, Julie Livingstone, Shadrielle Melijah G. Espiritu, Heather Thorne, Vincent Huang, Winnie Lo, Yu-Jia Shiah, Takafumi N. Yamaguchi, Ania Sliwinski, Sheri Horsburgh, Alice Meng, Lawrence E. Heisler, Nancy Yu, Fouad Yousif, Melissa Papargiris, Mitchell G. Lawrence, Lee Timms, Declan G. Murphy, Mark Frydenberg, Julia F. Hopkins, Damien Bolton, David Clouston, John D. McPherson, Theodorus van der Kwast, Paul C. Boutros, Gail P. Risbridger, Robert G. Bristow

**Affiliations:** 1 Monash Partners Comprehensive Cancer Consortium and Cancer Program, Biomedicine Discovery Institute, Department of Physiology, Monash University, Melbourne, Victoria 3800, Australia; 2 Princess Margaret Cancer Centre, University Health Network, Toronto, Ontario, Canada M5G 1L7; 3 Informatics & Biocomputing Program, Ontario Institute for Cancer Research, Toronto, Ontario, Canada M5G 0A3; 4 kConFab, Research Department, Peter MacCallum Cancer Centre, Melbourne, Victoria 3000, Australia; 5 The Sir Peter MacCallum Department of Oncology University of Melbourne, Parkville, Victoria 3010, Australia; 6 Department of Urology, University of Melbourne, Austin Hospital, Heidelberg, Victoria 3084, Australia; 7 Monash Partners Comprehensive Cancer Consortium and Cancer Program Biomedicine Discovery Institute, Department of Anatomy and Developmental Biology, Monash University, Melbourne, Victoria 3800, Australia; 8 Genome Technologies Program, Ontario Institute for Cancer Research, Toronto, Ontario, Canada M5G 0A3; 9 Department of Cancer Surgery, Peter MacCallum Cancer Centre, Melbourne, Victoria 3000, Australia; 10 Urological Pathology, Tissupath, Mt Waverley, Victoria 3149, Australia; 11 Department of Medical Biophysics, University of Toronto, Toronto, Ontario, Canada M5G 1L7; 12 Department of Pharmacology & Toxicology, University of Toronto, Toronto, Ontario, Canada M5S 1A8

## Abstract

Germline mutations in the *BRCA2* tumour suppressor are associated with both an increased lifetime risk of developing prostate cancer (PCa) and increased risk of aggressive disease. To understand this aggression, here we profile the genomes and methylomes of localized PCa from 14 carriers of deleterious germline *BRCA2* mutations (*BRCA2*-mutant PCa). We show that *BRCA2*-mutant PCa harbour increased genomic instability and a mutational profile that more closely resembles metastastic than localized disease. *BRCA2*-mutant PCa shows genomic and epigenomic dysregulation of the *MED12L*/*MED12* axis, which is frequently dysregulated in metastatic castration-resistant prostate cancer (mCRPC). This dysregulation is enriched in *BRCA2*-mutant PCa harbouring intraductal carcinoma (IDC). Microdissection and sequencing of IDC and juxtaposed adjacent non-IDC invasive carcinoma in 10 patients demonstrates a common ancestor to both histopathologies. Overall we show that localized castration-sensitive *BRCA2*-mutant tumours are uniquely aggressive, due to *de novo* aberration in genes usually associated with metastatic disease, justifying aggressive initial treatment.

## Abstract (web summary)

Men that carrier *BRCA2* germline mutations are at risk of developing prostate cancer. Here, the authors analyse the genomes of prostate cancer from these individuals and demonstrate increased genomic instability in comparison to sporadic prostate cancer.

Germline mutation of the *BRCA2* tumour suppressor gene substantially increases the lifetime risk of developing prostate cancer (PCa)^1^,^2^. In *BRCA2*-mutation carriers, localized PCa rapidly progresses to metastatic castrate-resistant prostate cancer (mCRPC) with 5-year cancer-specific survival rates of ∼50–60% (refs 1, 3, 4). *BRCA2*-mutant tumours also exhibit an increased frequency of intraductal carcinoma (IDC), a pathology that predicts adverse outcome in both familial^5^ and sporadic PCa^6^,^7^. Prostate tumours arising in men with an inactivating *BRCA2* germline mutation (*BRCA2*-mutant PCa) are uniquely aggressive, associated with younger age of onset, have higher rates of lymph node and distant metastasis, and increased mortality relative to sporadic, non-*BRCA2*-mutant disease^3^,^5^,^6^. Precision surgery or radiotherapy with curative intent inexplicably fails, with a rapid onset of incurable mCRPC in *BRCA2*-mutant PCa^7^,^8^,^9^. The molecular origins of the clinical aggressiveness of *BRCA2*-mutant PCa are unknown. Understanding them may allow better treatment decision-making; for example, given their DNA repair deficient genotype, these cancers may be amenable to treatment with PARP inhibitors, based on genetic synthetic lethality^10^,^11^,^12^.

In this study, to identify the drivers of aggression in *BRCA2*-mutant PCa, we characterized genomic alterations in localized PCa tumour specimens from 14 men carrying a deleterious germline mutant *BRCA2* allele using either whole-genome sequencing (WGS) and/or SNP array-based copy-number analyses (CNAs). We find that *BRCA2*-mutant PCa harbours a mutational profile more akin to that of mCRPC than localized sporadic PCa, and shows broad dysregulation of pathways associated with aggressive disease, including the *MED12*/*MED12L* axis. Micro-dissection and subclonal reconstruction shows distinct evolutionary trajectories and identifies a common precursor to IDC and adjacent invasive carcinoma. Taken together, these data demonstrate the genomic correlates underlying aggressive *BRCA2*-mutant PCa.

## Results

### The Mutational Landscape of *BRCA2*-mutant PCa

To address intra-prostatic and intra-lesion heterogeneity, we microdissected regions of invasive carcinoma (IC) and of IDC from five tumours, resulting in 19 PCa specimens (Supplementary Table 1; Supplementary Fig. 1).

**Figure 1:**
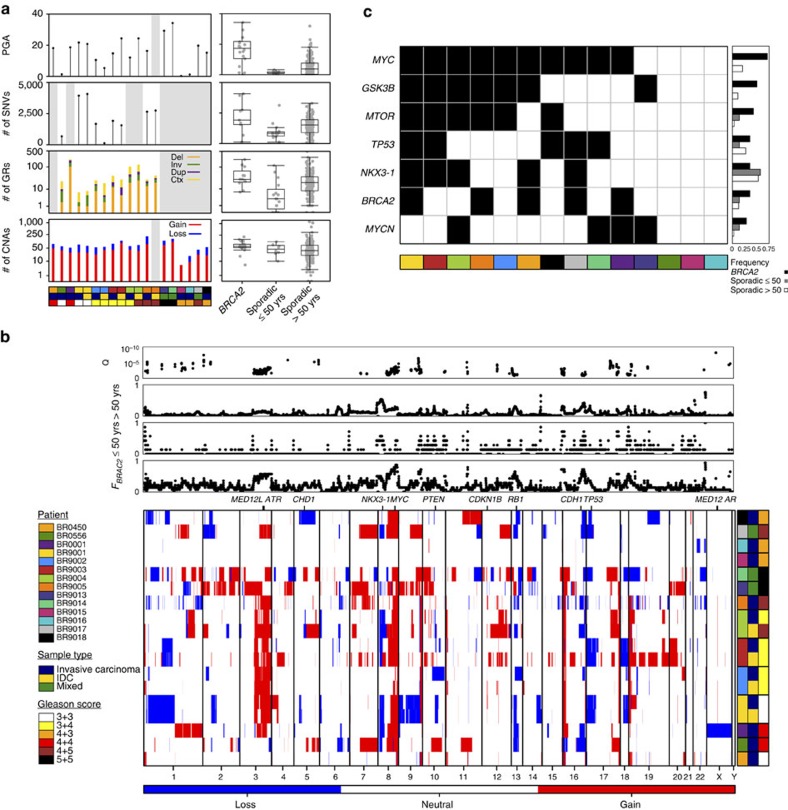
The genomics of *BRCA2*-mutant prostate cancer. (**a**) Percent genome altered (PGA) and number of SNVs, GRs and CNAs per tumour sample is shown. GRs are stratified by type: orange—deletions (del), green—inversions (inv), purple—duplications (dup), yellow—translocations (ctx). *Y* axis values for CNAs and GRs are in log_10_-scale. Box plots compare the number of events observed in *BRCA2*-mutant specimens versus sporadic PCa samples split into two groups by age. *Y* axis values are the same as in the adjacent bar plot. Whiskers indicate the maximum and minimum values, the box outline indicates the third and first quartile and the bar indicates the mean. Multiple foci from the same patient are indicated in the same patient covariate colour, where sample type colour indicates IC in blue, IDC in yellow and a mix of IDC and IC in green. Unavailable data is indicated by grey background. (**b**) Per gene copy-number profiles of 18 prostate tumour specimens from 14 germline *BRCA2*-mutation carriers, including 4 patients with multifocal disease. Red indicates gain; blue indicates loss. Rows represent specimens and columns represent genes. Top plots show frequency of each gene per group (*BRCA2*, *n*=14; sporadic PCa arising in individuals 50 years of age or younger, *n*=7; sporadic PCa arising in individuals older than 50 years of age, *n*=276) and the q-value shown is from a two-sided proportion test comparing *BRCA2*-mutant and all sporadic PCa samples. Genes are ordered by genomic co-ordinates per chromosome. Multiple foci from the same patient are indicated in the same patient covariate colour, where sample type colour indicates IC in blue, IDC in yellow and a mix of IDC and IC in green. (**c**) Key prostate cancer driver genes that are mutated at elevated proportions in *BRCA2*-mutant PCa relative to sporadic PCa. Columns are patients, rows are genes, a black square indicates a CNA. Bar plots to the right show the frequency of each gene in the three groups (BRCA2, *n*=14; sporadic PCa arising in individuals 50 years of age or younger, *n*=7; sporadic PCa arising in individuals older than 50 years of age, *n*=276).

We first identified single-nucleotide variants (SNVs), genomic rearrangements (GRs) and CNAs from these samples using validated pipelines (Supplementary Table 1; Supplementary Fig. 2). We compared the mutational profiles of *BRCA2*-mutant PCa to a set of 200 sporadic PCa with whole-genome sequencing^13^. We separated this comparison cohort into patients 50 years of age or younger at the time of treatment (*n*=8) and those older than 50 years (*n*=192), to control for age effects. *BRCA2*-mutant PCa showed elevated genomic instability, as measured by percent genome alteration (PGA), an established prognostic factor for relapse in localized disease (*P*=7.53 × 10^−4^; two-sided *t*-test; Fig. 1a)^14^,^15^. Similarly, *BRCA2*-mutant tumours showed elevated numbers of SNVs and GRs than sporadic tumours arising in either older or younger men (Supplementary Table 2; Supplementary Fig. 3).

Overall, *BRCA2*-mutant PCa shows the same C-class character^16^ as sporadic PCa, with a large number of CNAs and a relative paucity of SNVs. Regions of the genome recurrently gained or deleted in *BRCA2*-mutant PCa but not sporadic PCa include a large amplification on chromosome 3q (Fig. 1b). Genomic changes associated with aggressive disease were more common, including gain of the *MYC* oncogene, which occurred in 72% (13/18) of *BRCA2*-mutant PCa which was significantly higher than the 20.1% rate in sporadic PCa of equivalent Gleason score (57/283; *P*=1.42 × 10^−3^; two-sided proportion test^17^. Gains in *MYCN*, *GSK3B* and *MTOR* were also more common in *BRCA2*-mutant PCa. By contrast, losses of the tumour suppressors *TP53* and *NKX3-1* occurred at similar rates in *BRCA2*-mutant and sporadic PCa (Fig. 1c). Supplementary Data 1 lists all genes with differential rates of copy-number aberrations. No individual gene was altered by SNVs in more than one *BRCA2*-mutant patient (Supplementary Fig. 4). In fact, of the 11 most frequently mutated genes in a cohort of 477 sporadic localized PCa (ref. 13), only 1 was mutated in *BRCA2-*mutant PCa: *TP53* (Supplementary Fig. 5). Similarly of the 32 genes mutated in at least two *BRCA2*-mutant PCa, only 12 were mutated in 477 sporadic PCa. One *BRCA2*-mutant PCa harboured a kataegic event (Supplementary Fig. 6).

**Figure 2:**
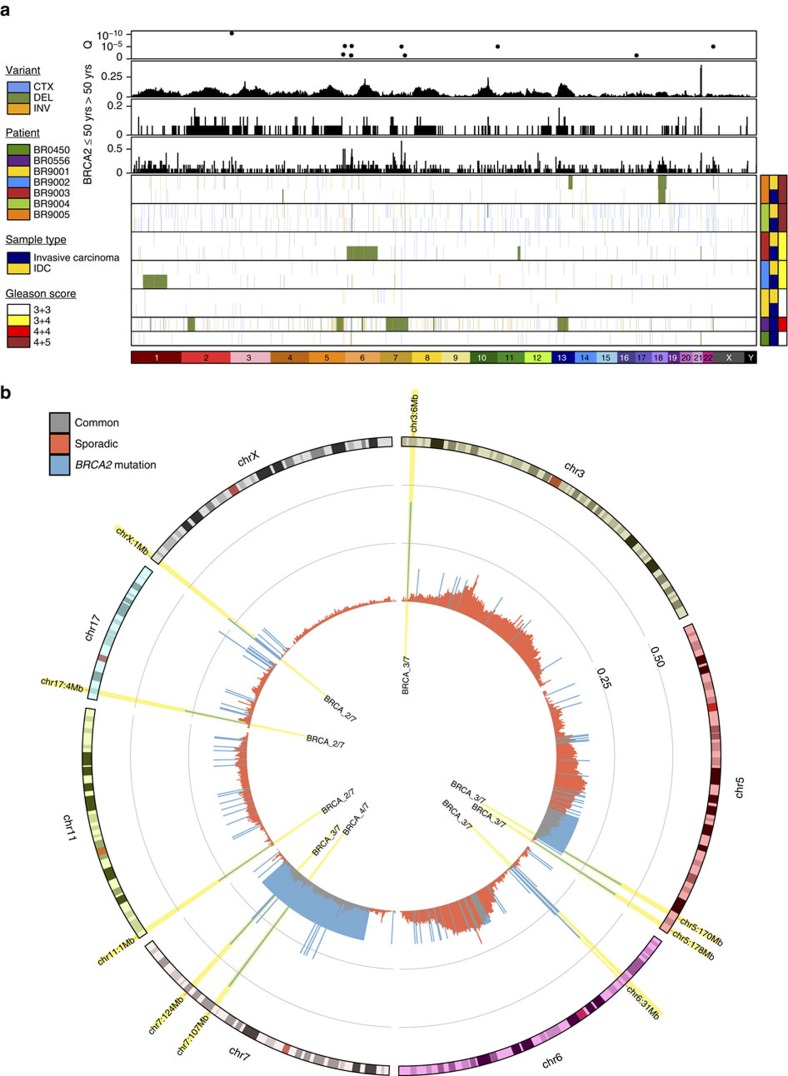
Genomic rearrangements in *BRCA2*-mutant prostate cancer. (**a**) Survey of genome-wide somatic genomic rearrangements (GRs) in *BRCA2*-mutant tumour PCa from seven patients. CTX—inter-chromosomal translocation; DEL—deletion; INV—inversion. Multiple foci from the same patient are indicated in the same patient covariate colour, where sample type colour indicates microdissected invasive carcinoma (IC) in blue and intraductal carcinoma of the prostate (IDC) in yellow. Top bar plots show the frequency of specimens in *BRCA2*-mutant PCa (bottom) and sporadic PCa from men 50 years of age or younger (middle) or sporadic PCa from men older than 50 years of age (top). Scatterplot at top indicates FDR-corrected *P* values of a two-sided proportion test comparing frequency of GRs observed in *BRCA2*-mutant versus sporadic PCa. (**b**) A circos plot showing the proportions of *BRCA2*-mutant (blue) and sporadic PCa (red) with breakpoints in each mega-basepair bin. Bins with significantly different proportions between *BRCA2*-mutant and sporadic PCa (FDR-correct proportions test) are highlighted in yellow, and have the number of *BRCA2*-mutant samples indicated. Only chromosomes with at least one differentially recurrent GR are shown.

Although the *TMPRSS2*:*ERG* gene fusion on chromosome 21 was observed at similar frequencies in *BRCA2*-mutant and sporadic PCa (Fig. 2a; 3/12 versus 68/200; *P*=0.74, proportion test; Supplementary Table 1), we identified several GRs in *BRCA2*-mutant PCa (Fig. 2b; Supplementary Fig. 7; Supplementary Data 2). The most prominent of these was an inversion on 3p26.1 which occurred in 4/12 *BRCA2*-mutant specimens but 0/200 sporadic PCa. This region contains only four genes: a metabotropic glutamate receptor (*GRM7*) and two of its antisense RNAs, along with a mitochondrial ribosomal pseudogene. *GRM7* inactivation suggests loss of negative regulation of NMDA signaling, as previously associated with the neuroendocrine disease (NED) phenotype. NED commonly arises in late-stage disease and is associated with treatment resistance and poor prognosis^18^. There was also a recurrent translocation on chromosome 7q in a non-genic region proximal to several solute carriers and membrane proteins. *BRCA2*-mutant PCa tumours showed similar chromothriptic character to sporadic disease (Supplementary Fig. 8).

Surprisingly, *BRCA2*-mutant PCa showed global hypomethylation relative to sporadic PCa (Supplementary Fig. 9) which was retained even after controlling for the increased CNA rate of *BRCA2*-mutant PCa (Supplementary Figs 10 and 17). Overall ∼1% (4,979/435,377) of copy-number neutral probes showed differential methylation between *BRCA2-*mutant and sporadic PCa specimens (Supplementary Data 3), and 74% (3,677/4,979) of these were hypomethylated, including 21 of the 25 most differentially methylated genes (Supplementary Fig. 11). Differentially methylated genes in *BRCA2*-mutant PCa were enriched in pathways associated with VEGF signaling, neuronal development, and other neuronal processes characteristic of NED (Supplementary Fig. 12; Supplementary Table 3). Other hypomethylated NED-related genes include modulators of WNT signaling (for example, *AXIN*, *APC*, *ROCK1/2*), IL6 signaling (for example, *IL6ST*, *BMP6*), and mTOR signaling (for example, *TSC2*), and many of these genes were also altered by CNAs (Supplementary Fig. 13). The 3′ UTR of the *BRCA2* locus was hypomethylated in *BRCA2*-mutant PCa specimens (Supplementary Fig. 14).

### MED12/MED12L amplification is associated with aggressive PCa

**Figure 3:**
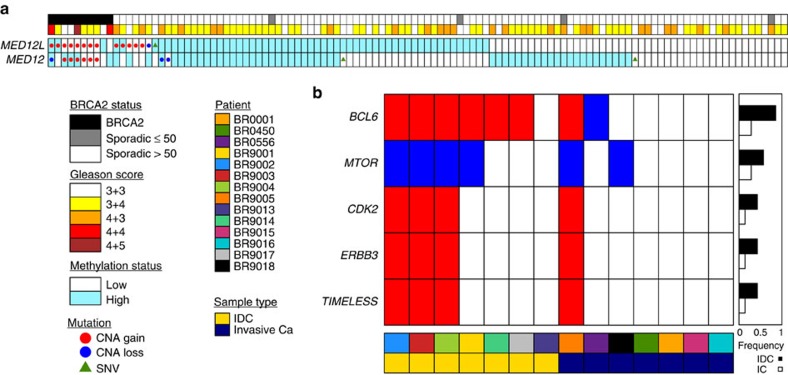
*BRCA2*-mutant PCa harbours multiple hallmarks of aggressive disease. (**a**) CNA, SNV and methylation profiles for *MED12L* and *MED12*. Patients are sorted based on *MED12L* CNA, methylation and SNV status. β-values are median dichotomized, as reflected by the background shading of each cell. The covariate bar at the top of the graph indicates *BRCA2* status and Gleason score. (**b**) Multiple genes show differential rates of CNAs in tumours harbouring IDC and those not. Rows are genes, columns are samples. Red indicates copy-number gain; blue indicates copy-number loss; white indicates neutral copy-number status. The barplot to the right shows the differential frequency of mutation between IDC and IC components in *BRCA2*-mutant PCa.

To identify other driver events that might help explain the aggressiveness of *BRCA2*-mutant PCa, we investigated the amplification of a region on chromosome 3q (Fig. 1b) that is common in *BRCA2*-mutant PCa (11/18; 61%), but rare in sporadic PCa (55/809; 6.8% *P*=1.64 × 10^−9^, two-sided proportion test). The consensus amplicon of this locus contains the WNT/β-catenin pathway modulator *MED12L* (Supplementary Data 4 and 7), as well as *ATR*, which encodes the DNA damage response factor ataxia telangiectasia-and-Rad3 related protein. Importantly, the *MED12L* homolog, *MED12* (chromosome Xq13.1), is amplified in 44% of *BRCA2*-mutant PCa specimens (8/18) compared with only ∼0.1% of localized sporadic PCa (1/809, *P*=3.64 × 10^−44^, two-sided proportion test). Further, *MED12L* (but not *MED12*) showed frequent amplification in distant metastases from metastatic castrate resistant prostate cancer (mCRPC) patients (7/37 cases, 19%). Supporting this model, four loci within the *MED12L* gene were significantly hypomethylated in *BRCA2*-mutant versus sporadic PCa (Fig. 3a; Supplementary Fig. 15). Consistent with their alteration at the copy-number and methylation levels in aggressive *BRCA2*-mutant PCa, *MED12*/*MED12L* are also amplified and/or show elevated RNA expression levels in 18% (26/150) of mCRPC^6^ but only 7.5% (25/333) of sporadic, localized PCa^18^ (*P*=0.002, proportion test). Thus *MED12L/MED12* amplifications are preferentially mutated in aggressive disease—both *BRCA2*-driven primary tumours and sporadic mCRPC.

*MED12L*/*MED12* amplification was also enriched in *BRCA2*-mutant PCa harbouring IDC (6/8 IDC-positive specimens with *MED12L*/*MED12* gain versus 1/6 IDC negative specimens). Importantly, these mutations are not enriched in sporadic tumours harbouring IDC. Therefore we evaluated global differences between IDC and IC. Presence of IDC in *BRCA2*-mutant PCa associates with CNAs portending poor prognosis. Several genomic regions were preferentially amplified or deleted in tumours harbouring IDC, including gain of *BCL6* and loss of *MTOR*, along with gains of a region harbouring *CDK2* and *ERBB3* (Fig. 3b). This underpins the observation that the presence of IDC in *BRCA2*-mutant PCa associates with an aggressive clinical course and increased PCa-specific mortality rates^5^.

### Evolutionary trajectories of *BRCA2*-mutant and sporadic PCa

**Figure 4:**
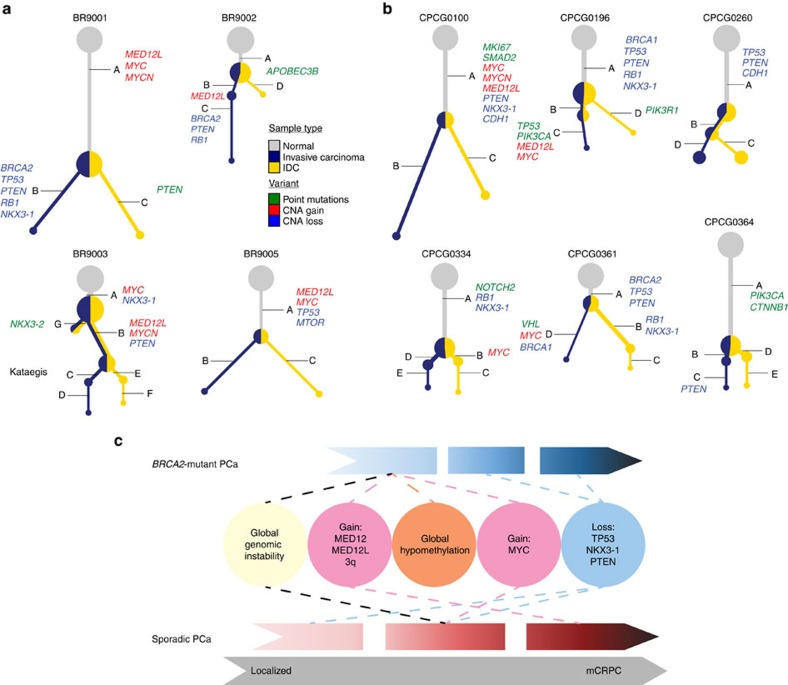
Evolutionary trajectory of *BRCA2*-mutant and sporadic PCa harbouring IDC. (**a**) Subclonal reconstruction of four *BRCA2*-mutant PCa from microdissected IDC and IC components using CNA and SNV data. (**b**) Reconstructions of six sporadic PCa with microdissected IDC and IC components using CNA and SNV data. Each tree gives the inferred phylogeny for a single patient. The gray node is the germline. Blue nodes are present in the IC specimen, gold nodes in the IDC specimen, and the relative proportions of each reflect their relative cellular prevalence. Node size reflects the sum of the cellular prevalence of the IDC and IC components. A set of key mutations that occur on each branch are annotated, and the length of the branch is proportional to the number of SNVs that accumulated on it. (**c**) Early in the development of *BRCA2*-Mutant PCa, the disease is characterized by amplifications in 3q, *MED12*, *MED12L* and *MYC*, global genomic instability, a global hypomethylation profile and presents as a more aggressive disease. Tumour suppressors appear to be lost early in sporadic PCa, leading to subsequent development of global genome instability, and increased amplifications as the disease advances.

Enrichment for negative prognostic factors in *BRCA2*-mutant PCa harbouring IDC suggested that these tumours may have distinct evolutionary trajectories. To assess the subclonal architectures of sporadic and *BRCA2*-mutant PCa harbouring IDC we selected six sporadic PCa and microdissected the IDC and IC components (Supplementary Fig. 16). Both components were subjected to whole-genome sequencing and CNA profiling and tumour phylogenies were reconstructed. This procedure was repeated for four *BRCA2*-mutant PCa cases that harboured both IDC and IC components. Reconstruction began with subclonal CNA profiling using TITAN^19^, followed by clustering of SNV frequencies with PhyloWGS^20^. In all four *BRCA2*-mutant PCa (Fig. 4a; Supplementary Data 5) the IDC and IC components arose from the same founding clone, with no evidence of multiple independent tumours^21^. The parental population was found in both the IDC and IC regions, providing no inference as to where the tumour may have initiated. *MYC* amplifications were observed in three of these four patients, and in each occurred before divergence of IDC and IC components. By contrast, *MED12L* gain was clonal in two samples and sub-clonal in two others, in one case only occurring in the IC component.

In sporadic PCa with evidence of IDC (Fig. 4b; Supplementary Data 6) there was again no evidence of multiple independent tumours—the IDC and IC components arose from a common ancestor and there was no clear evidence as to which compartment this ancestor arose in. *MYC* amplification was observed in 4/6 tumours, once clonally, once in a clone only present in IDC and twice only in a clone present in IC. *NKX3-1* deletion was present in the trunk of 3/6 tumours. Intriguingly, *PIK3CA* SNVs were observed in 2/6 patients with IDC (once clonally and once only in the IC component), as compared with 7/333 patients in the TCGA localized PCa dataset (*P*=5.92 × 10^−4^; proportion test)^9^.

## Discussion

While the clinical aggressiveness of germline *BRCA2*-mutant PCa remains unexplained, it is distinguished from sporadic PCa by a unique mutational profile. *BRCA2*-mutant PCa is characterized by elevated global genomic instability, which is a well-established biomarker of biochemical failure and metastasis^14^,^15^. *BRCA2*-mutant PCa harboured specific mutations that are rarely, if ever, observed in sporadic localized PCa. These lead to dysregulation of pathways associated with NED and mCRPC, despite no exposure to androgen deprivation therapy in these patients. These observations help rationalize our recent clinical association between disease outcome and the presence of IDC^5^, in part, with increased activation of the *MED12L*/*MED12* axis. Surprisingly, and counter to previous expectations, the IDC and IC components share a common precursor, and share a large portion of their mutational profile.

*MED12L/MED12* are components of the mediator complex and important regulators of the WNT pathway, which has been implicated in cell proliferation and neuroendocrine differentiation in PCa^22^,^23^,^24^,^25^. The *MED12L*/*MED12* axis is implicated in metastatic sporadic PCa, with *MED12* nuclear overexpression common in mCRPC and localized recurrent PCa but not in localized primary PCa or benign prostate tissue^26^. *MED12* SNVs, which occur in both localized and advanced PCa, are also implicated in prostate tumourigenesis^27^ and genome instability^28^,^29^.

*BRCA2*-mutant PCa are also enriched for aberrant DNA methylation in genes involved in neurogenesis and neuron development, consistent with recent reports that autonomic nerve development contributes to PCa progression and metastasis^30^. Overall, our data strongly support a model in which germline *BRCA2*-mutant PCa are genotypically-similar to mCRPC and frequently harbour genomic aberrations in key pathways (for example, WNT/APC, IL6-BMP6, mTOR, ATR, MYCN, and so on; Supplementary Data 6; Fig. 4c) leading to genomic instability, NED, castrate-resistance and metastasis, together explaining adverse patient prognosis. It remains to be shown if the temporal change in genomic stability of *BRCA2*-mutation carrier PCa tumours is relevant to other solid tumours with specific pre-dispositions for aggressive disease such as those arising in breast, colon and pancreas.

Collectively, our data provide genome-wide, nucleotide-resolution characterization of the molecular architecture of localized PCa and IDC that develops in men who harbour a mutant germline *BRCA2* allele. While the population frequency is relatively low (<1%)^31^, the aggressive natural history of localized tumours may justify a change in clinical management to a more intensified approach including upfront use of PARP inhibitors^10^ and chemotherapy (for example, success of CHAARTED, STAMPEDE trials)^32^ in addition to ADT to block the rapid progression to mCRPC observed in these patients. Based on our provocative data, trials could also explore molecular inhibition of ATR, mTOR and GSK3A pathways in these patients, especially when IDC is present. Our results may ultimately help to elucidate multiple key pathways to therapeutically target men who are carriers of a *BRCA2* mutation with PCa to increase rates of cure by preventing rapid progression to mCRPC.

## Methods

### Patient cohorts

Men were treated for sporadic or *BRCA2*-mutant PCa by radical prostatectomy at Princess Margaret Cancer Centre (University Health Network, Toronto, Canada) or were recruited by kConfab (Kathleen Cuningham Foundation Consortium for research into Familial Breast cancer) at the Peter MacCallum Cancer Centre (Melbourne, Australia). Informed consent for analysis was obtained through institutional Research Ethics Board protocols (UHN #11-0024 and #11-0091-T; #97/27).

In Canada, patients with a known BRCA2 germline mutation were approached for retrospective informed consent based on the following inclusion criteria: (1) intermediate or high risk localized Prostate cancer, as defined by NCCN guidelines, treated in the Princess Margaret Cancer Centre BRCA clinic; (2) confirmed presence of primary tumour tissue in the UHN genitourinary biobank; (3) whole blood (5 ml) was obtained at the time of consenting. Consent was for use of tumour and normal tissues and associated DNA, RNA and protein, including whole-genome and whole-transcriptome sequencing of both somatic and germline specimens.

In Australia, prostate cancer cases were identified from 1,600 families recruited into the Kathleen Cunningham Consortium for Research into Familial Breast Cancer (kConFab), the Australian and New Zealand consortium for families at high-risk of breast cancer (http://www.kconfab.org). Inclusion in kConFab requires a strong multi-case family history of breast and/or ovarian cancer, or a germline mutation in a breast cancer predisposition gene such as BRCA1and BRCA2 (see www.kconfab.org for full recruitment criteria). Male kConFab members were eligible for inclusion in this study if: (i) they had a verified diagnosis of prostate cancer, (ii) complete diagnostic and treatment notes were available and (iii) family BRCA mutation status was known. Human Research Ethics Committee approval (protocol: 97/27) was obtained from Peter MacCallum Cancer Centre to allow recruitment of prostate cancer patients at the time of diagnosis for fresh tissue collection or access to retrospectively archived formalin fixed paraffin embedded tissues. A comparative cohort of patients with sporadic prostate cancer was recruited for pathology review; these men were spouses of the multi-case breast cancer women recruited into kConFab.

### Tissue collection and microdissection

Hematoxylin and Eosin-stained sections from fresh-frozen or formalin-fixed paraffin-embedded (FFPE) prostate cancer tissues were assessed by a clinical genitourinary pathologist (TvdK or DC). Regions of at least 70% tumour cellularity were manually microdissected from fresh-frozen or FFPE tissues from *BRCA2* or sporadic tissues.

### Copy-number profiling

Genome-wide gene CNA profiles were generated with Affymetrix OncoScan v3 SNP microarrays. Genomic DNA was extracted from microdissected tumour specimens using phenol:chloroform, as previously described^23^), and quantified using a Qubit Fluorometer. Microarray analysis used 30–150 ng of dsDNA for each sample. Analysis of Affymetrix OncoScan FFPE Express 3.0 SNP probe assays was performed using. OSCHP files generated by OncoScan Console 1.1 using a custom reference when starting material was Fresh Frozen (*n*=12). A custom reference which included 119 normal blood samples from male patients with prostate cancer, 2 normal blood samples from females with anaplastic thyroid cancer and 10 female HapMap cell line samples was created to combat artefacts created due to the difference in sample preparation (FFPE versus Fresh Frozen).

For FFPE samples,. OSCHP files generated by Affymetrix using their in-house reference (*n*=8).

BioDiscovery's Nexus Express for OncoScan 3 Software were used to call CNAs with the SNP-FASST2 algorithm. Default parameters were used, except that the minimum number of probes per segment was changed from 3 to 10. When necessary, samples were re-centered using the Nexus Express software, choosing regions that showed diploid log_2_ratio and B allele frequency profiles (*n*=2). Gene level copy-number aberrations for each patient were identified by overlapping CN segments with RefGene annotation using BEDTools (v2.17.0)^33^. Tumour purity was assessed computationally (Supplementary Fig. 15). PGA was calculated for each sample by dividing the number of base-pairs that are involved in a copy-number change by the total length of the genome.

To discover differences in copy-number states between germline *BRCA2* mutated samples (*n*=14) and sporadic samples (*n*=284) two-sided proportion tests were performed as implemented in R (v3.1.3). A ‘by bvote' profile was created for patients with both IDC and IC samples. When the copy-number state per gene differed between samples, gain and deletions were kept over neutral states. Copy-number segment data was mapped to the RefGene annotation, classifying each gene's state as ‘amplification, ‘deletion' or ‘neutral' and then amplifications and deletions were grouped together for this analysis. *P* values were FDR adjusted to account for multiple testing. Two-sided, unpaired *t*-tests were performed to test differences in PGA between *BRCA2* and sporadic samples implemented in R (v3.1.3).

The copy-number states of specific prognostic genes were investigated to determine if the frequency differed between samples that had an IDC component and those that did not. Samples that had both IDC and IC specimens were combined to get a ‘complete' profile, in which the union of all aberrations was taken. Only CNAs that are in the same state (amplification versus deletion) as known prognostic genes are shown. For example if a specimen has amplification of *NKX3-1*, it will not be shown, as only *NKX3-1* deletions have prognostic value. A Wilcoxon test was performed comparing the rank sum of CNAs in three prognostic genes (that is, *NXK3-1*, *MYC*, *PTEN*) between patients with IDC and those without.

### Whole-genome sequencing

Whole-genome sequencing was performed on Illumina HiSeq 2,500 instruments at the Ontario Institute for Cancer Research. Sequencing libraries were prepared on 150 ng of input genomic DNA, as previously reported^21^. Tumour and normal DNA were sequenced to target depths of 50 × and 30 × , respectively (actual sequencing depths shown in Supplementary Table 1).

### Whole-genome sequencing data analysis

Each lane of raw sequencing reads was aligned against human reference build GRCh37 with decoy (hs37d5) using bwa (v0.7.11)^34^. Lane level BAMs from the same library were merged, marking duplicates using picard (v1.92). Library level BAMs from each sample were merged without marking duplicates. The Genome Analysis Toolkit (GATK v2.4.9) was used for local realignment and base quality recalibration, processing tumour/normal pairs together^35^. Separate tumour and normal sample level BAMs were extracted, headers were corrected using samtools (v0.1.9)^36^ and files were indexed with picard (v1.107). To evaluate cross-individual contamination, ContEst (v1.0.24530) was used for all the normal and tumour sequences^37^. Both the sample and lane-level analysis were performed and confirmed no significant contamination. Regarding the required input VCFs, genotype information was gained from the germline SNVs generated by GATK (v2.4.9) and the VCF for population allele frequencies for each SNP in HapMap (hg19) was downloaded from https://www.broadinstitute.org/cancer/cga/contest_download.

Somatic SNVs were predicted using SomaticSniper (v1.0.2)^38^. First, somatic SNV candidates were detected using bam-somaticsniper with the default parameters except -q option (mapping quality threshold). The -q was set to 1 instead of 0 as recommended by the developer. To filter the candidate SNVs, a pileup indel file was generated for both normal BAM and tumour BAM file using SAMtools (v0.1.6). The SomaticSniper package provides a series of Perl scripts to filter out possible false positives (http://gmt.genome.wustl.edu/somatic-sniper/1.0.3/documentation.html). First, standard and LOH filtering were performed using the pileup indel files and then, bam-readcount filter was also performed (bam-readcount downloaded on 10 January 2014) with a mapping quality filter -q 1 (otherwise default settings). In addition, we ran the false positive filter. Subsequently, a high confidence filter was used with the default parameters. The final VCF file that contains high confidence somatic SNVs was used in the downstream analysis.

After somatic SNV calling using SomaticSniper, identified SNVs were passed through an annotation pipeline. SNVs were functionally annotated by ANNOVAR (v2015-06-17)^39^, using the RefGene database (downloaded on 2014-07-15). Nonsynonymous, stop-loss, stop-gain and splice-site SNVs (based on RefGene annotations) were considered functional. If more than one mutation is found in a sample for a gene, then the mutation of the higher priority functional class was used for visualization. SNVs were filtered using tabixpp (3b299cc0911debadc435fdae60bbb72bd10f6d84), removing SNVs found in any of the following databases: dbSNP141 (modified to remove somatic and clinical variants, with variants with the following flags excluded: SAO=2/3, PM, CDA, TPA, MUT and OM)^40^, 1,000 Genomes Project (v3), Complete Genomics 69 whole genomes, duplicate gene database (v68)^41^, ENCODE DAC and Duke Mapability Consensus Excludable databases (comprising poorly mapping reads, repeat regions, and mitochondrial and ribosomal DNA)^42^, invalidated somatic SNVs from 68 human colorectal cancer exomes (unpublished data) using the AccuSNP platform (Roche NimbleGen), germline SNVs from 477 sporadic PCa patients with the intermediate GS (3+X and X+3) and additional 10 prostate cancer patients with higher GS^13^, and the Fuentes database of likely false positive variants^43^. SNVs were whitelisted (and retained, independently of the presence in other filters) if they were contained within the Catalogue of Somatic Mutations in Cancer (COSMIC) database (v70)^44^.

### SNV mutation load

To find the total number of bases somatic SNVs can be called on, each position in the reference was classified as ‘callable' or not. Positions were considered callable in a sample if it has a minimum coverage of 10 × in normal and 17 × in the tumour as calculated using BEDTools (v2.18.2)^33^. Mutation load for each sample was then calculated as the number of somatic SNVs divided by the total number of callable bases and converted to per mega-basepair of sequence. Mutation load was not calculated for samples missing a normal specimen to avoid upwards bias on estimated mutation rates.

### Genomic rearrangements

For each patient, aligned tumour and normal BAM files were used to call structural variants with Delly (v0.5.5)^45^ at a minimum median mapping quality of 20 and a paired-end cut-off of five. A list of somatic variants were produced by removing germline mutations from the resulting VCF files, which were further filtered using a consolidated list of structural variants from 124 normal samples. To identify genes affected by the genomic rearrangements, bed files were generated for each sample from deleted regions, and breakpoints from inversions, inter-chromosomal translocations, and tandem duplications. The resultant bed files were examined with SnpEff (v3.5)^46^ and gene names were subsequently extracted for downstream analyses. Recurrent translocation events were visualized using Circos (v0.67-4)^47^. Input files were bed files containing paired translocation breakpoints and the number of samples the event was observed. Differentially aberrant regions between sporadic and *BRCA2* patients were identified by first dividing the genome into mega-basepair bins, and tabulating the number of samples with a deletion, translocation or inversion breakpoint in each bin. Subsequently, a difference in proportions test was performed for each bin and the resultant *P* values were corrected for multiple comparisons with FDR. Entire chromosomes containing mega-basepair bins with significantly different proportions of breakpoints were visualized using Circos (v0.67-4)^47^.

### Chromothripsis detection

ShatterProof^48^ was used with default settings to examine the presence of chromothripsis through the analysis of copy-number aberrations, loss of heterozygosity, and genomic translocations, and duplications. Samples with a maximum ShatterProof score of at least 0.517 were defined as having chromothriptic characteristics.

### Subclonal reconstruction

For each of the four *BRCA2*-mutant PCa and six sporadic PCa subjects with microdissected IDC and IC components, phylogenetic trees were constructed using the predicted SNVs and CNAs inferred by TITAN^19^. TITAN was run, from a range of one to five clonal clusters, on each sample and the CNA segments associated with the run having lowest *S_Dbw* validity index was used for further analysis. Using the predicted SNVs, a multi-sample PhyloWGS^20^ run was conducted on each subject to cluster the SNV frequencies. PhyloWGS Witness was used to view and determine the consensus phylogenetic tree from the many sampled trees, summarizing the co-evolution of IDC and IC (Fig. 4; Supplementary Data 5 and 6) from the normal sample (gray). For each tree, blue denotes IC while gold denotes IDC, and the relative proportions of each reflect their relative cellular prevalence. Node size reflects the sum of the cellular prevalence of the IDC and IC components. Length of the branch is proportional to the number of SNVs that accumulated on it, while its width is scaled to the cellular prevalence of the new arising node. Validated pipelines identified recurrent mutations across the samples and using the gene list associated with the top PhyloWGS tree, key SNV mutations (green) were annotated along the branches. With OncoScan runs as a reference, TITAN's subclonal CNAs and their inferred cellular prevalence identified a set of key driver genes, both gains (red) and losses (blue) along these branches, whereas parsimony was assumed.

### Methylation microarray data analysis

Illumina Infinium HumanMethylation 450 k BeadChip kits were used to assess global methylation, using 500 ng of input genomic DNA at the McGill University and Genome Quebec Innovation Centre (Montreal, QC). This study comprises 10 *BRCA2*-mutant and 104 sporadic samples from 8 processing batches. All methylation analysis was performed in R statistical environment (v3.2.1). The IDAT files were loaded and converted to raw intensity with the use of the wateRmelon package (v1.6.0)^49^ from the BioConductor (v3.0) open-source project. Quality control was conducted using the minfi package (v1.12.0)^50^ (no outlier samples were detected). Batch effects were also examined across 5 batches (BRCA2 versus sporadic samples) using the mclust package (v5.0.2) and no batch effects were found (Adjusted Rand Index=0.070). Raw methylation intensity levels were then pre-processed using Dasen. Probe filtering was conducted after the normalization. For each probe, a detection p-value is computed to indicate whether the signal for the corresponding genomic position is distinguishable from the background noise. Probes having 1% of samples with a detection p-value greater than 0.05 were removed (2,816 probes). We also filtered probes based on SNPs (65 probes) and non-CpG methylation probes (2,944 probes). Next, we used the DMRcate package (v1.2.0)^51^ to further filter out 27,296 probes that are known to cross-hybridize to multiple locations in the genome and 17,079 probes that contain a SNP with an annotated minor allele frequency of greater than 5% with a maximum distance of two nucleotides to the nearest CpG site. Average intensity levels were taken for technical replicates. Probes showing differential methylations between BRCA and sporadic samples (age>50 years) were identified using the DMRcate package. Probes were deemed to be significantly differentially methylated based on *q*<0.05, |log_2_ FC| of β-values>0.1, and CNA status. The fraction of patients having a CNA event (whether it is a gain or loss) is calculated and only probes with fraction CNA<0.1 were kept. Next, probes were annotated to their respective gene symbols via IlluminaHumanMethylation450kanno.ilmn12.hg19 (v0.2.1) annotation package, resulting in 2,624 unique genes; this gene list showing differential methylations were analysed using g:GOSt in the g:profiler web database (build: 2014-04-11)^52^. Significantly enriched pathways (*P*<0.05; hypergeometric test), as an output from g:profiler^52^, were then visualized using Cytoscape (v3.3.0)^53^. Tumour purities for all the samples were assessed with LUMP^54^.

### Public data analysis

To assess the occurrence of MED12/MED12L amplifications in sporadic and mCRPC relative to *BRCA2*-mutant cancers, we downloaded publically available gene copy-number data from TCGA^9^ (*n*=203), Baca^55^ (*n*=57), Barbieri^56^ (*n*=109) and Taylor^8^ (sporadic, *n*=157; mCRPC, *n*=37). To assess the relationship between RNA expression and MED12L/MED12 amplification, we queried cBioPortal for the presence of RNA over-expression (z-score>±2.0) and amplification of one or both of these genes in two studies for which both RNA-seq and CNA data were available: Robinson *et al*.^7^ (mCRPC) and TCGA^9^ (localized sporadic PCa). A case was counted as positive if either RNA was overexpressed or the gene was amplified.

### Data availability

Raw sequencing and SNP microarray data from this study is available at EGA under the study accession EGAS00001001615. Methylation data from this study is available at GEO under accession GSE80685. Methylation data accessed from GEO with the accession code GSE84043 was used to support the findings of this study. Sequencing data accessed from EGA with the accession codes EGAS000010000258 and EGAS000010000900 and from dbGaP with the accession codes phs000447.v1.p1 and phs000330.v1.p1 was used to support the findings of this study. A subset of the TCGA-PRAD dataset was also downloaded from CGHub (data now stored at the Genomic Data Commons). The authors declare that all remaining data are contained within the Article and its Supplementary Information files or available from the author upon request.

## Additional information

**How to cite this article:** Taylor, R. A. *et al*. Germline *BRCA2* mutations drive prostate cancers with distinct evolutionary trajectories. *Nat. Commun.* **8,** 13671 doi: 10.1038/ncomms13671 (2017).

**Publisher's note**: Springer Nature remains neutral with regard to jurisdictional claims in published maps and institutional affiliations.

## Supplementary Material

Supplementary Information Supplementary Figures 1-17 and Supplementary Tables 1-3.

Supplementary Data 1 Genes that are copy number altered at differential frequencies between BRCA2-mutant (n = 14) and sporadic (n = 283) cases. Two-sided proportion tests were performed per gene and p-values were adjusted with FDR to account for multiple testing.

Supplementary Data 2 List of genes within mega-basepair bins that have significant differentially abundant genomic rearrangements between the BRCA2-mutant (n = 12) and sporadic PCa (n = 200) as calculated using a difference in proportions test and corrected using FDR.

Supplementary Data 3 4,979 significantly differentially methylated probes (q < 0.05, |log2 beta FC| > 0.1; CNA fraction < 0.1) between BRCA2-mutant and sporadic PCa. A linear model was used to assess significant differential methylation patterns. β-FC represents the log2 fold change of the β-values between the two patient groups. P-values were multiple-testing adjusted with False Discovery Rate (FDR) algorithm.

Supplementary Data 4 Genes on chromosome 3 that are amplified at higher frequencies in BRCA2-mutant vs. sporadic PCa. Two-sided proportion tests were performed per gene and p-values were adjusted with FDR to account for multiple testing.

Supplementary Data 5 The truncal and branch information of four BRCA2-mutant PCa are summarized in this table. Columns 3 and 4 highlight the cellular prevalence of tumours harbouring IC and IDC components, respectively, that arise at the end of that branch.

Supplementary Data 6 The truncal and branch information of six sporadic PCa are summarized in this table. Columns 3 and 4 highlight the cellular prevalence of tumours harbouring IC and IDC components, respectively, that arise at the end of that branch. Column 5 counts the number of SNVs along the branch while column 6 lists the genes that accumulated SNVs. Columns 7 and 8 list the genomic coordinates (format gene: chromosome: start-end) of altered genes resulting from copy number changes.

Supplementary Data 7 CNA status of 18 BRCA2-mutant specimens. First 5 columns contain hg19 gene annotation information (RefGene ID, gene symbol, chromosome, start position, end position). Columns starting with BR contain gene level copy number data per specimen, where 0 indicates neutral, 1 indicates copy number gain and -1 indicated copy number deletion.

Peer Review File
