# Supplementary material for: Germline BRCA2 mutations drive prostate cancers with distinct evolutionary trajectories
Source: Nat Commun. 2017 Jan 9;8:13671. doi: 10.1038/ncomms13671 (PMC5227331; doi:10.1038/ncomms13671)
Supplement: Supplementary Information — Supplementary Figures 1-17 and Supplementary Tables 1-3. [file ncomms13671-s1.pdf]

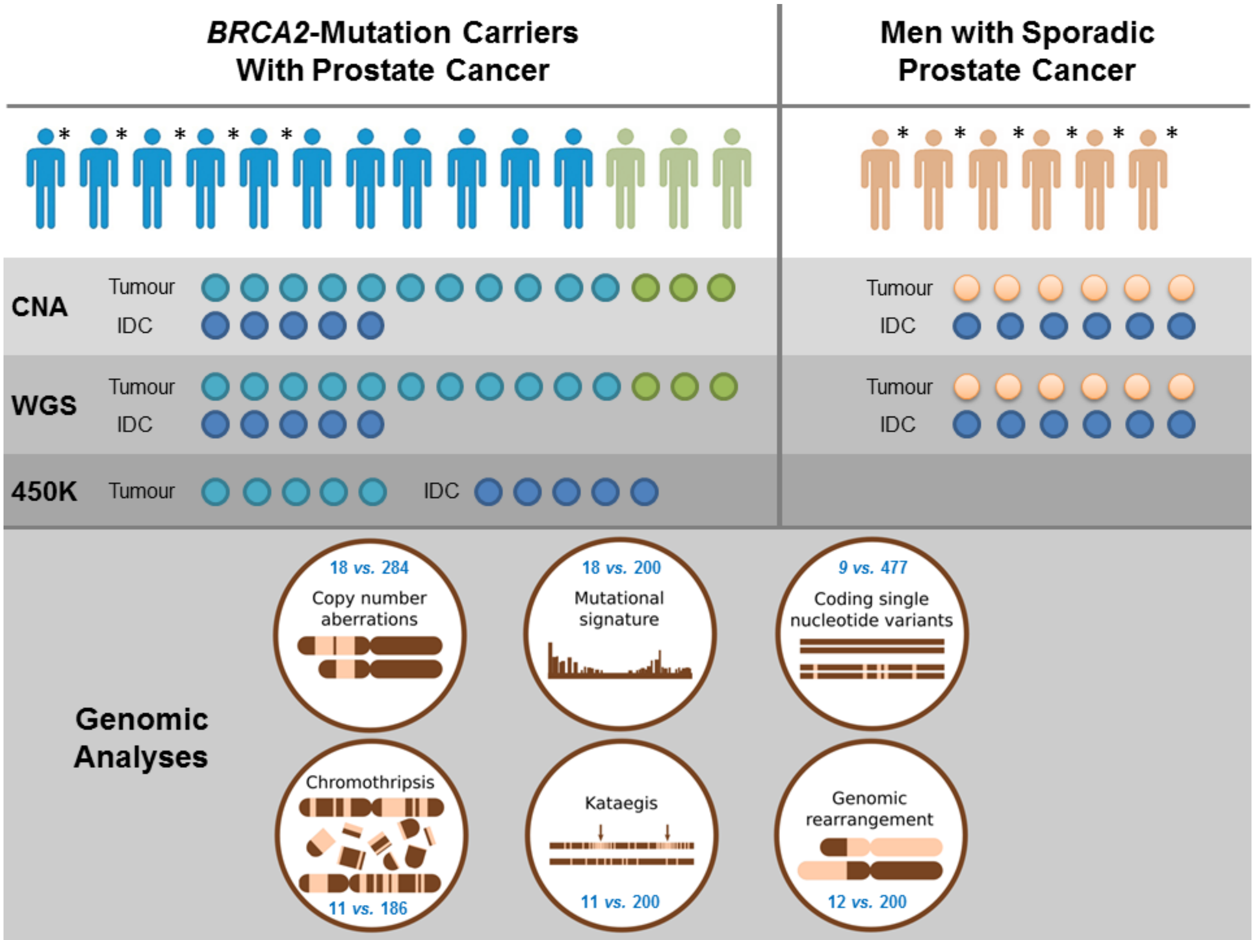

Supplementary Figure 1 | Overall study design

We obtained 19 tumour specimens from 14 men with prostate cancer who harboured pathogenic germline *BRCA2*-mutations. Germline DNA was available for five patients and for 11/14 patients, we obtained isolated invasive carcinoma (IC) tissue (IC; represented by blue men). Intraductal carcinoma (IDC) was micro-dissected from five of these 11 patient specimens (represented as asterisks). For the remaining 3/14 patients, specimens were obtained from mixed IC and IDC pathologies (represented as the green men). We subjected 18/19 specimens to copy number profiling (one specimen failed QC) and 12/19 specimens were subjected to whole genome sequencing (WGS; plus germline DNA from five patients). Further, 10/19 specimens had methylome interrogation (5 IC, 5 IDC). In addition, IC and IDC were also micro-dissected from six men with sporadic (*i.e.* non-familial) PCa (orange). These additional 12 sporadic PCa specimens (in addition to blood-derived germline DNA) were also subjected to whole-genome sequencing and CNA analysis. We conducted analyses and comparisons to publically available data from localized, sporadic prostate cancers. For CNAs, we compared 18 *BRCA2*–mutant PCa specimens (14 IC, 4 IDC) to 284 sporadic PCa. For mutational signatures, we compared 18 *BRCA2*-mutant PCa specimens (14 IC, 4 IDC) to 200 sporadic PCa specimens. For single nucleotide variant analysis in gene coding regions, we compared nine *BRCA2*-mutant PCa specimens (5 IC, 4 IDC) to 477 sporadic PCa. For chromothripsis and kataegis analysis, we compared 11 *BRCA2*-mutant PCa specimens (3 IDC, 8 IC) to 186 and 200 sporadic PCa specimens, respectively. For genomic rearrangements, we compared 12 (7 IC, 5 IDC) *BRCA2*-mutant PCa specimens to 200 sporadic PCa specimens.

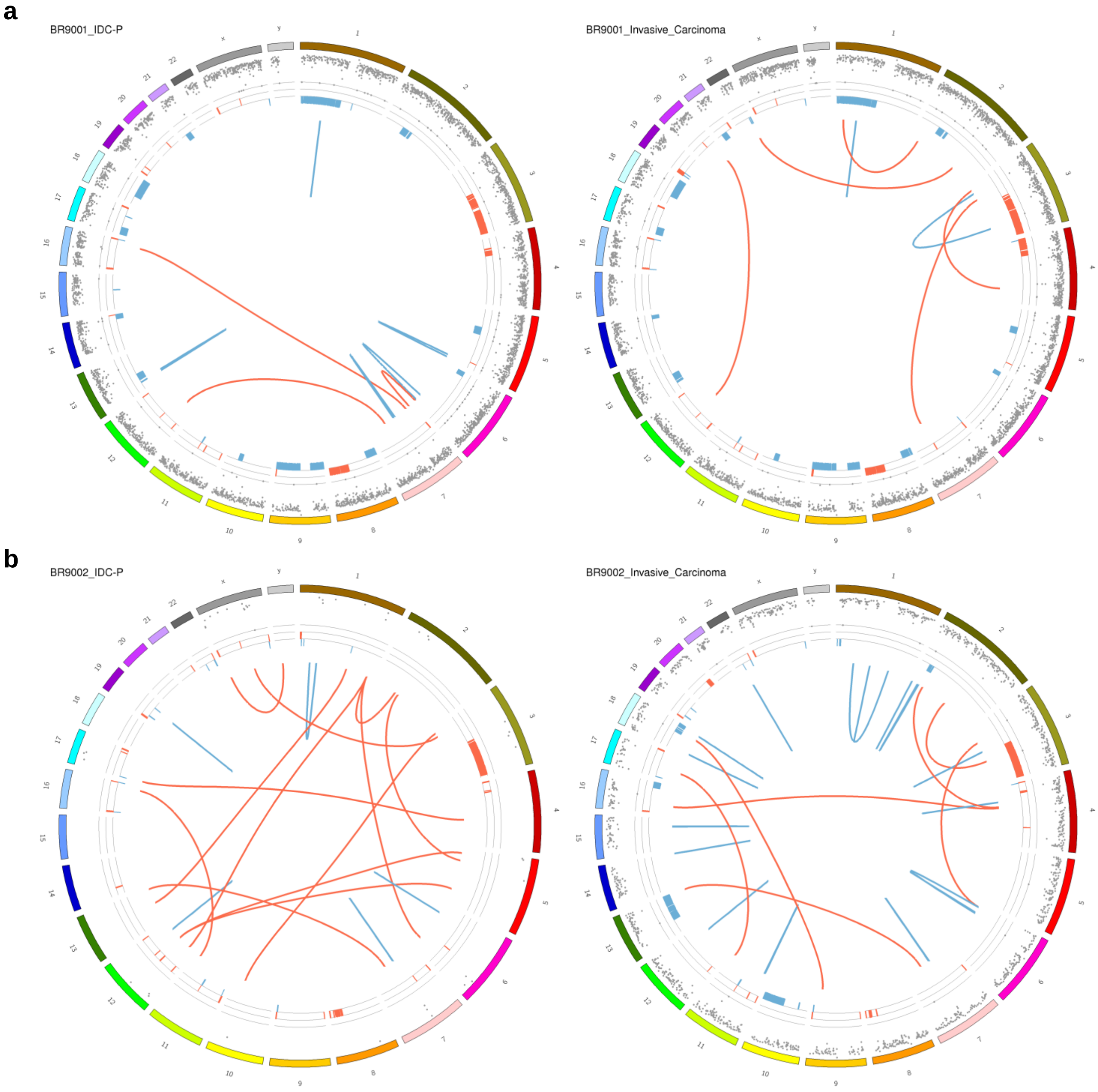
a
BR9001_IDC-P
BR9001_Invasive_Carcinoma
b
BR9002_IDC-P
BR9002_Invasive_Carcinoma

c

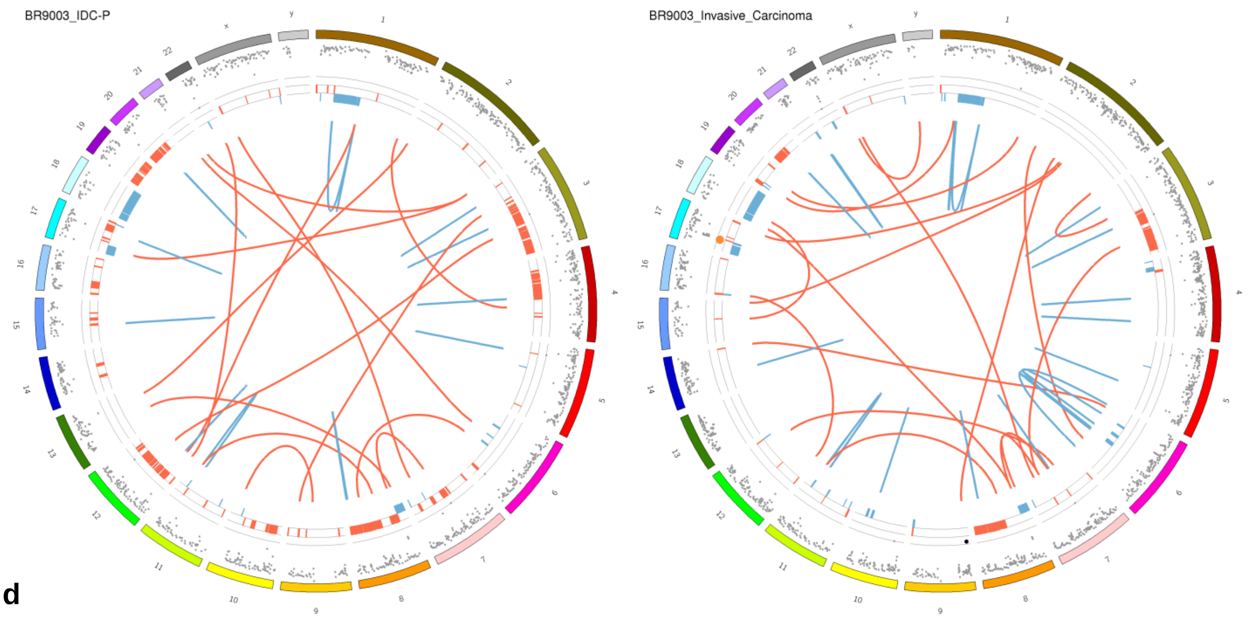
BR9003_IDC-P
BR9003_Invasive_Carcinoma

d

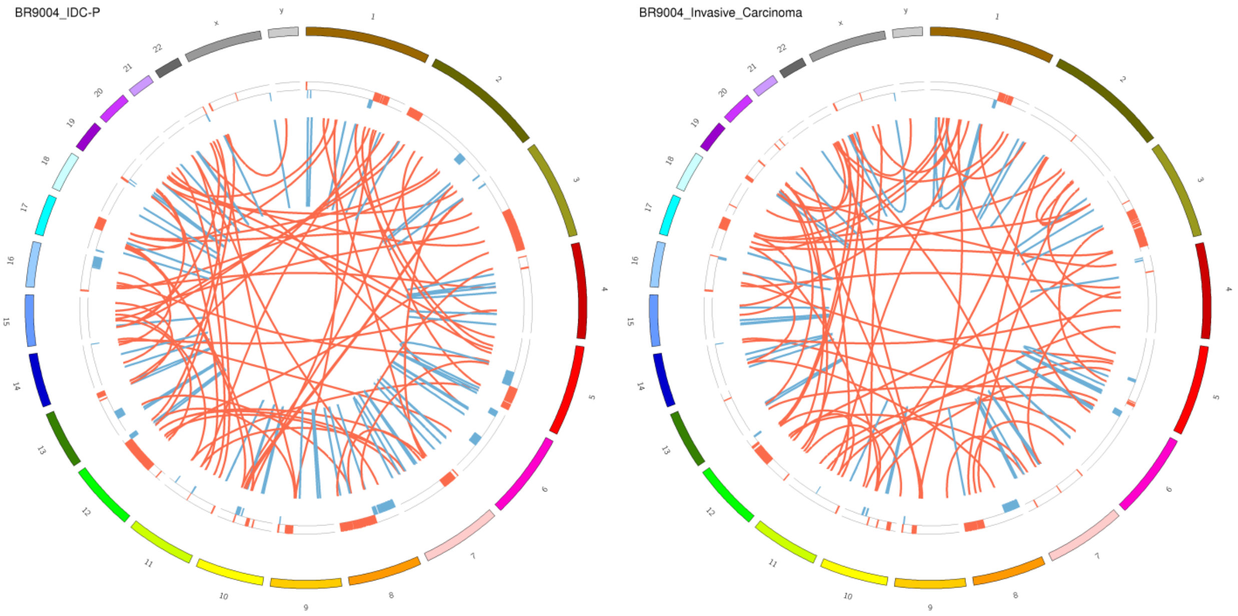
BR9004_IDC-P
BR9004_Invasive_Carcinoma

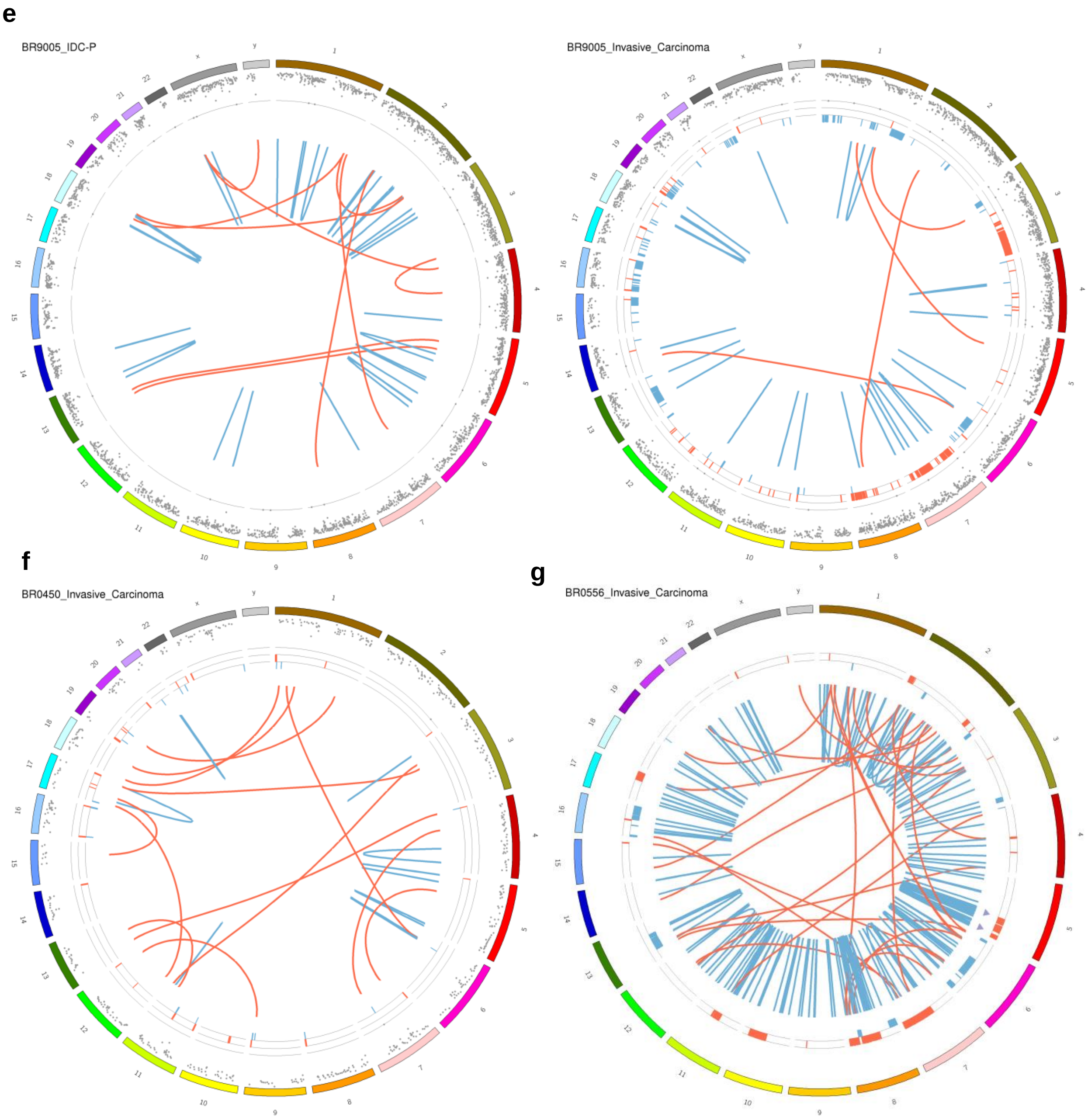
e
BR9005_IDC-P
BR9005_Invasive_Carcinoma
f
BR0450_Invasive_Carcinoma
g
BR0556_Invasive_Carcinoma

h

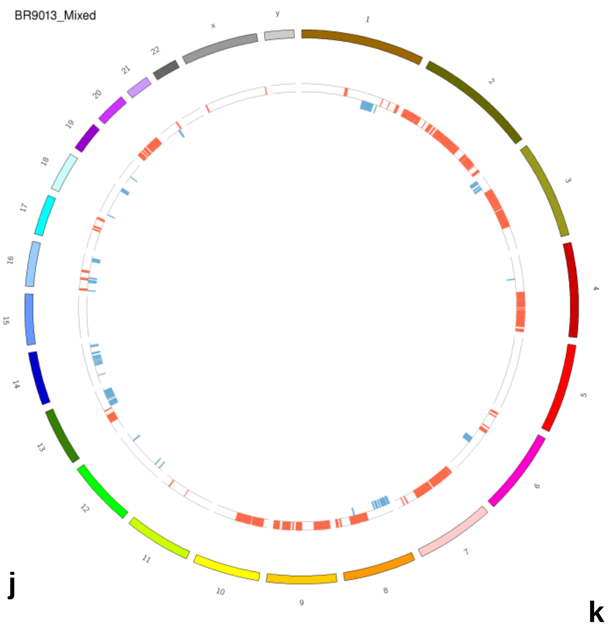
BR9013_Mixed

i

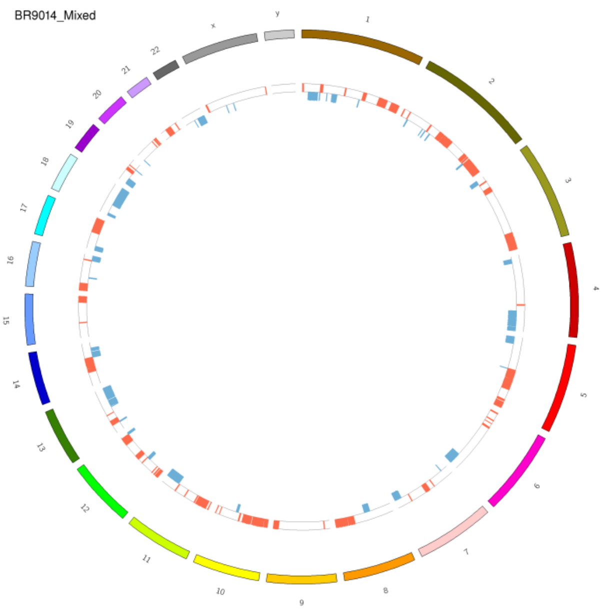
BR9014_Mixed

j

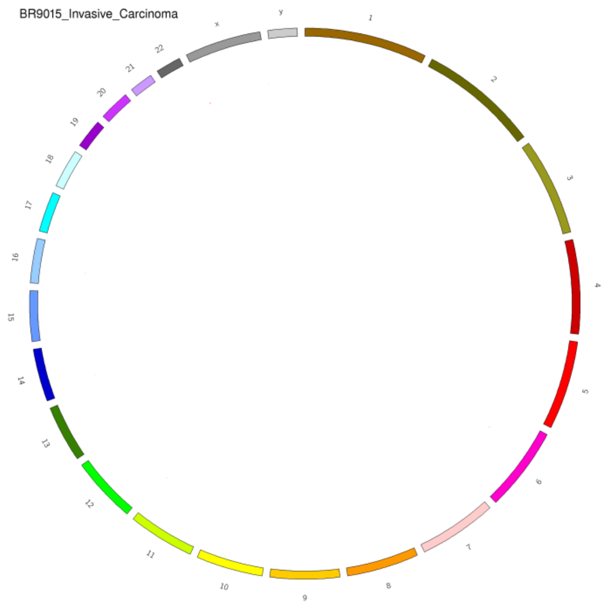
BR9015_Invasive_Carcinoma

k

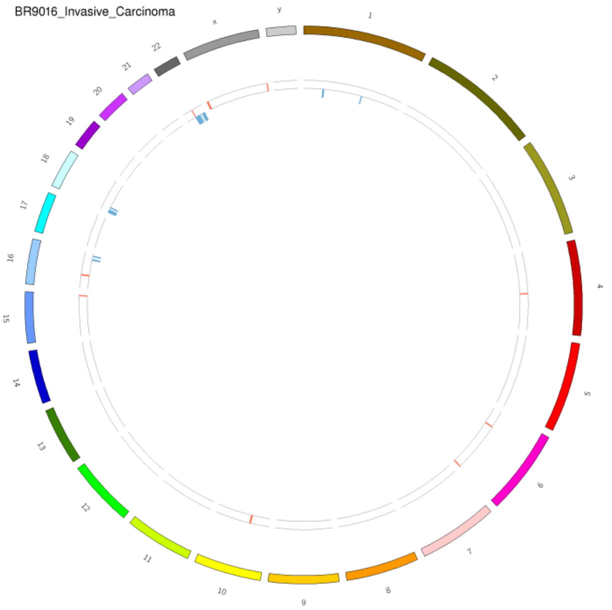
BR9016_Invasive_Carcinoma

l

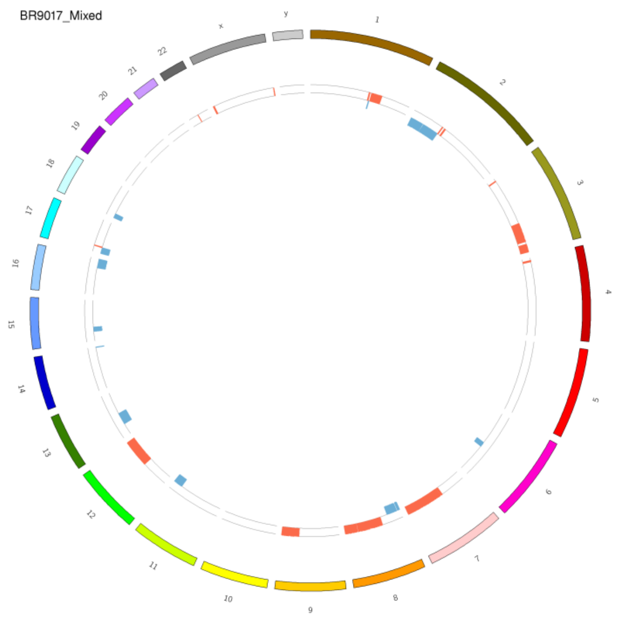

m

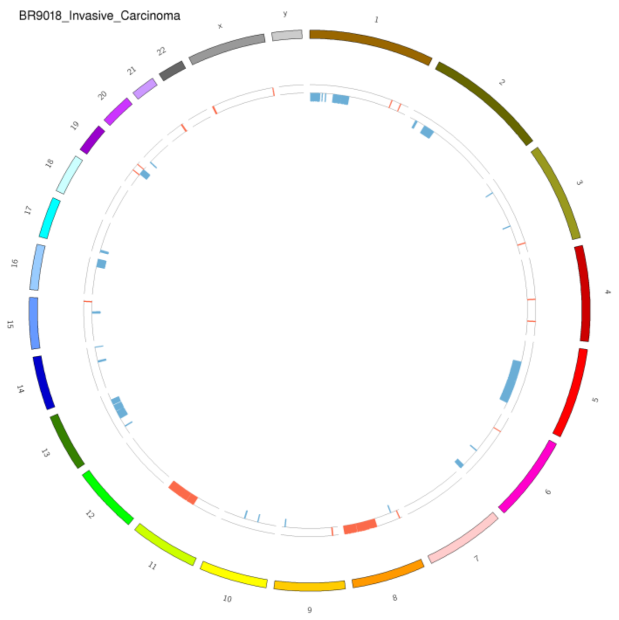

Supplementary Figure 2 | Overall mutation profile of each tumour

(a-m) Circos plots indicate (from outside-in): chromosome number, scatterplot of SNV inter-mutational distance (distance of each somatic variant to the somatic variant adjacent to it), kataegis events (black indicates a significant hypermutation region, orange indicates that the hypermutation contains a C/TAG enrichment), CNAs (red indicates amplifications, blue indicates deletions), chromothripsis events (purple triangles), inter-chromosomal translocation (orange lines) and inversions (blue lines).

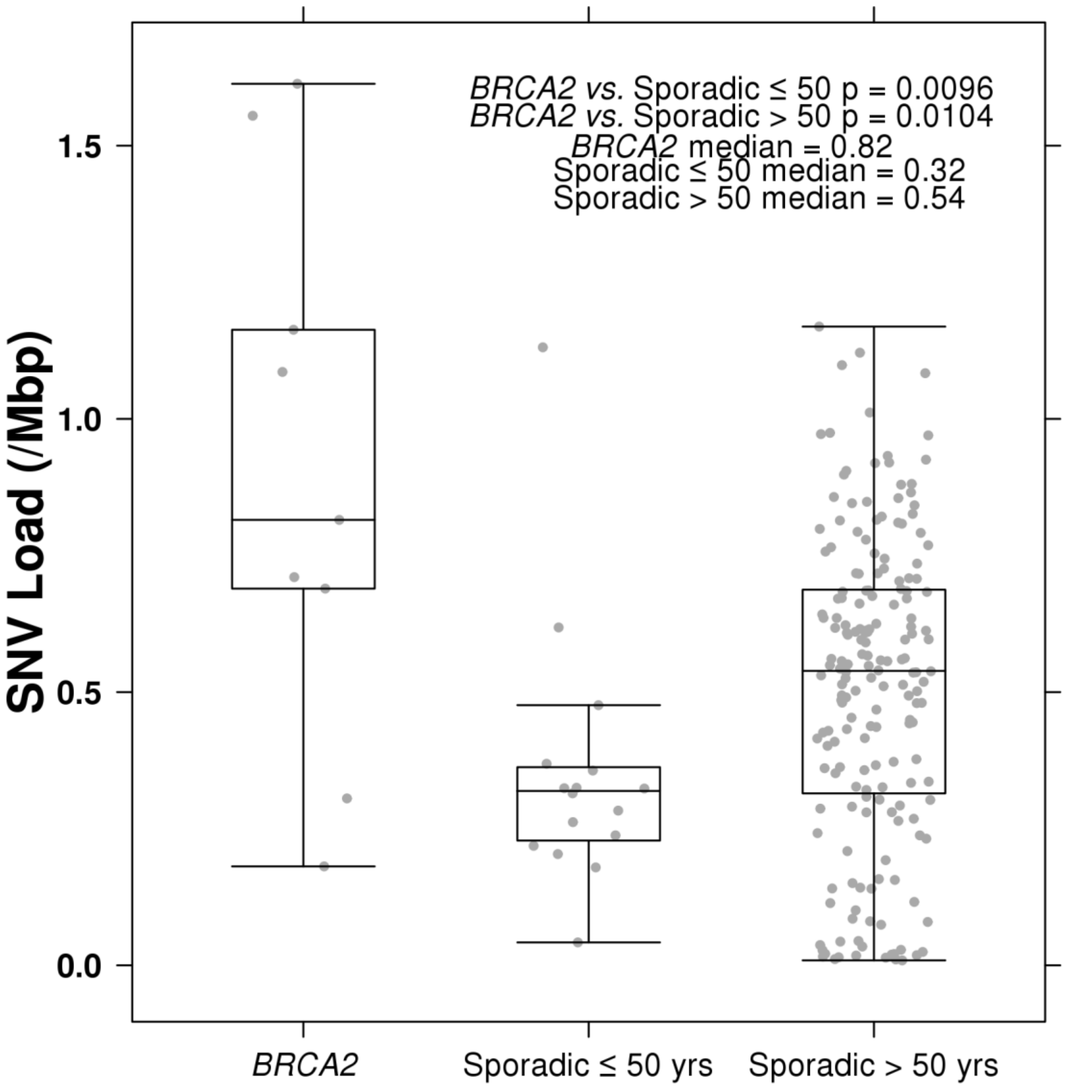

Supplementary Figure 3 | SNV mutation density

SNV mutation density (somatic SNVs per mega-basepair of DNA sequenced) is compared between *BRCA2*–mutant PCa and sporadic PCa. Sporadic PCa are stratified by patient age at initial treatment. P-values were calculated using a Mann-Whitney U-test. Whiskers indicate the maximum and minimum values, the box outline indicates the third and first quartile and the bar indicates the mean.

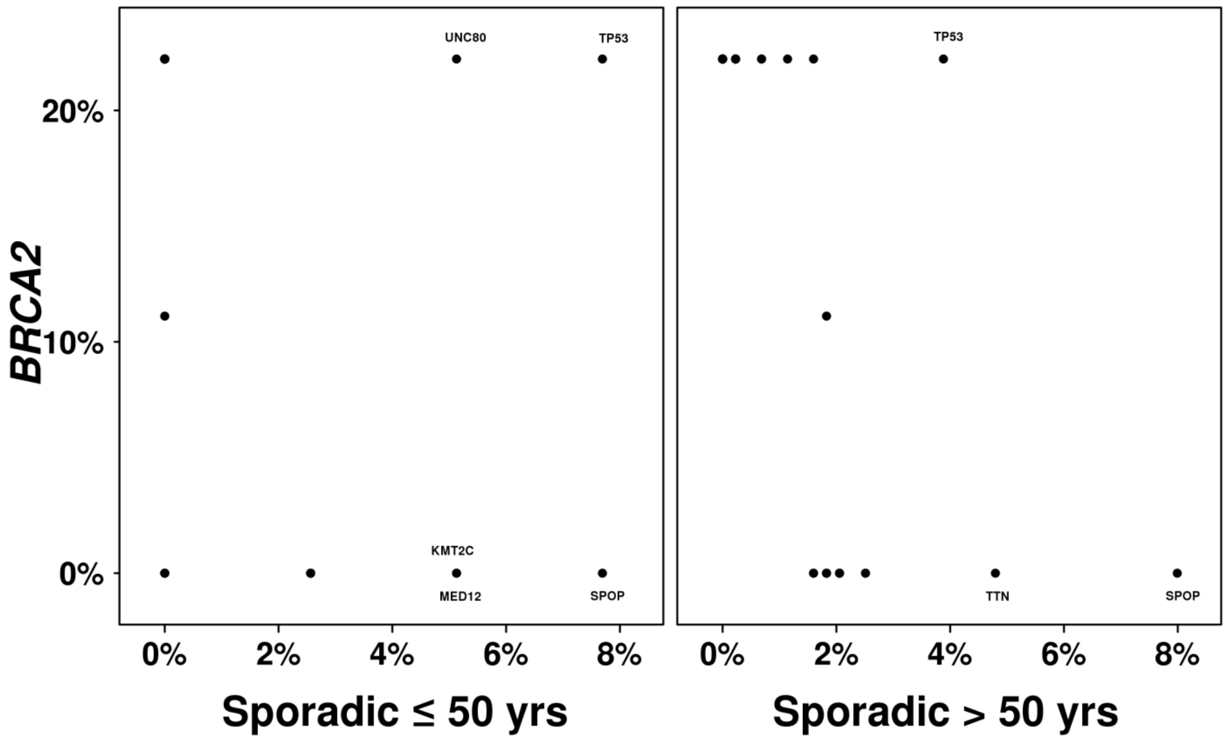

Supplementary Figure 4 | Genes altered by SNVs in *BRCA2*-mutant and sporadic PCa

Relative aberration frequencies of genes recurrently disrupted by SNVs in localized *BRCA2*-mutant (n = 9) *vs.* sporadic PCa (n = 477). Genes were considered recurrently altered if found in at least eight sporadic or two *BRCA2*-mutant specimens. Sporadic samples were split into two groups based on age at initial treatment; 50 years of age or younger (left) and older than 50 years of age (right). Some points are occupied by multiple genes with identical occurrence frequencies.

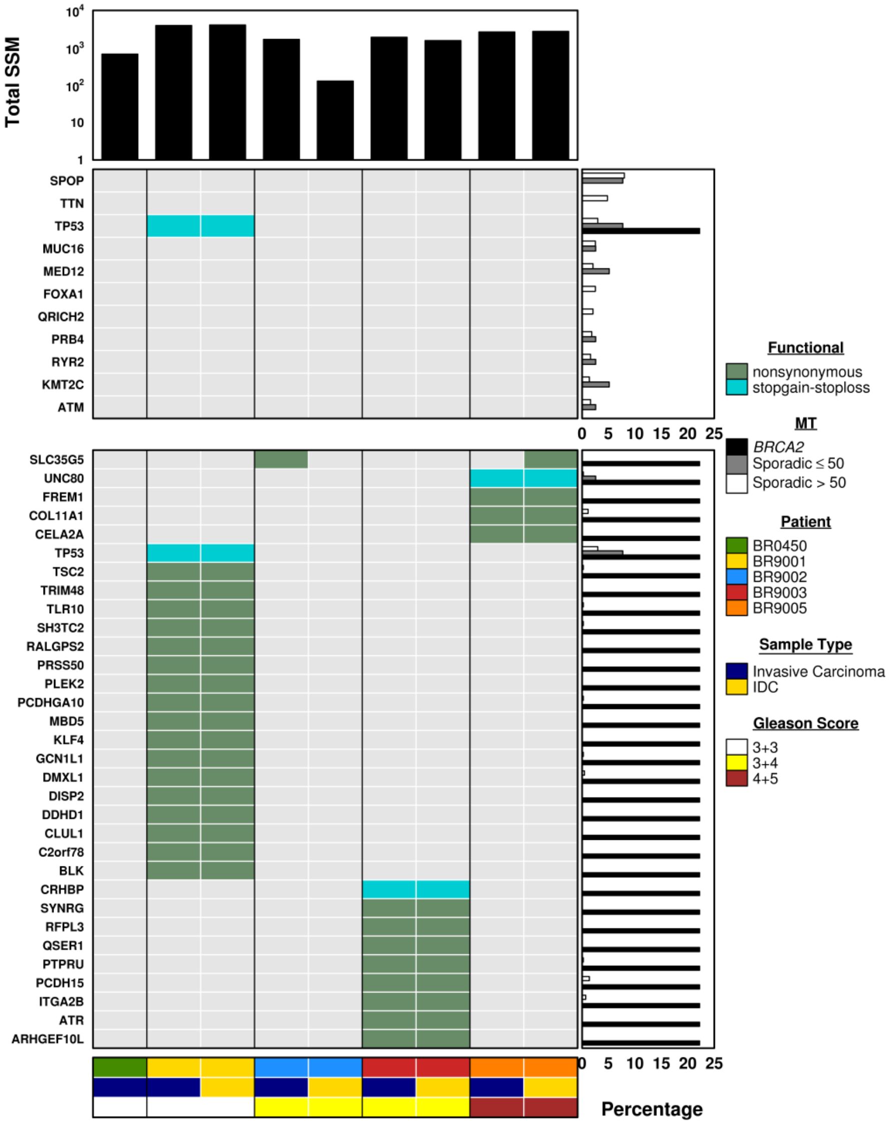

Supplementary Figure 5 | Comparison of somatic SNV profile between *BRCA2*-mutant and sporadic PCa

Somatic SNV predictions in protein-coding regions from five *BRCA2*-mutant samples were compared to recurrently mutated genes in sporadic PCa (n = 477). The top barplot shows the total number of single nucleotide variants (SNV) per specimen. The central heatmap shows the recurrent somatic SNV predictions in the coding regions for the five *BRCA2*-mutant cancers. Tumours are sorted by patient id, sample type and Gleason score (bottom covariates). The top central heatmap shows overlap between the top 11 recurrently mutated genes, each found in at least eight specimens, detected in the sporadic cases and the recurrent genes found in the *BRCA2*–mutant cases. The percentage of recurrence found in sporadic cases 50 years of age or younger (n = 39), sporadic cases older than 50 years of age (n = 438), or in *BRCA2*-mutant cases are shown at the right side of each plot. Multiple foci from the same patient are indicated in the same patient covariate colour, where sample type colour indicates invasive carcinoma (IC) in dark blue and intraductal carcinoma (IDC) in yellow.

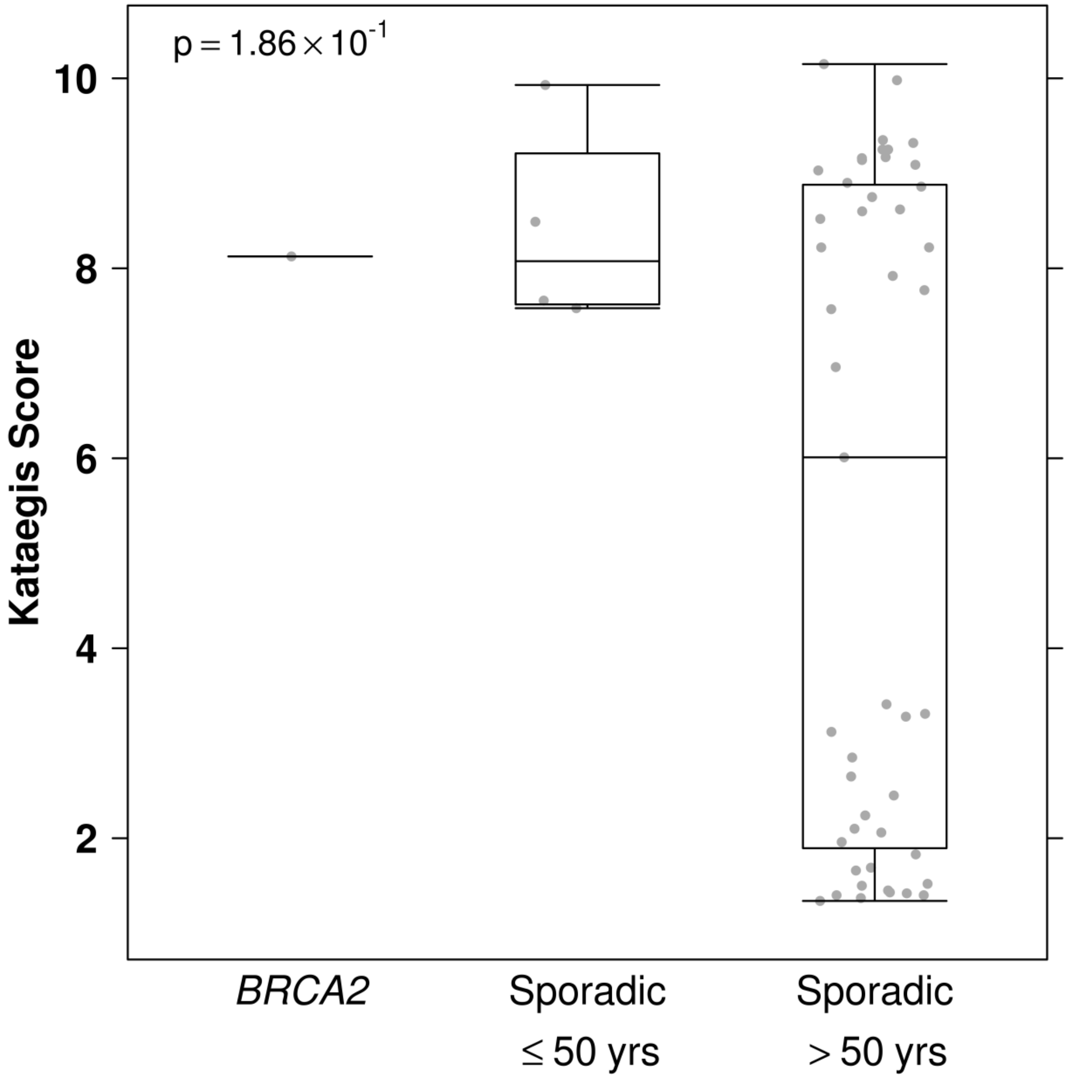

Supplementary Figure 6 | Kataegis in *BRCA2*-mutant and sporadic PCa

Boxplot comparing kataegis scores, generated by SeqKat, between *BRCA2*-mutant (n=11) and sporadic (n=133) PCa specimens. Values are shown for specimens, only if a kataegis even was identified. P-value is from a one-way ANOVA. Whiskers indicate the maximum and minimum values, the box outline indicates the third and first quartile and the bar indicates the mean.

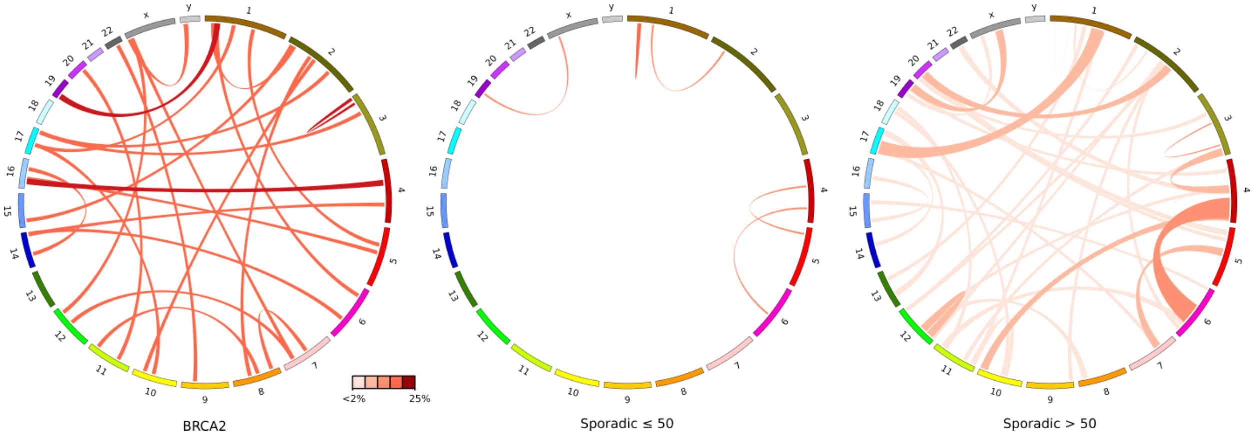

Supplementary Figure 7 | Genomic rearrangements in *BRCA2*-mutant and sporadic PCa

Circos plots illustrating translocation and inversion events in *BRCA2*-mutant PCa (left) and sporadic PCa from men 50 years of age and younger (center) and men older than 50 years of age (right). The percentage of specimens the event was observed in is represented by the colour and thickness of the lines. Light red < 2%, dark red > 25%.

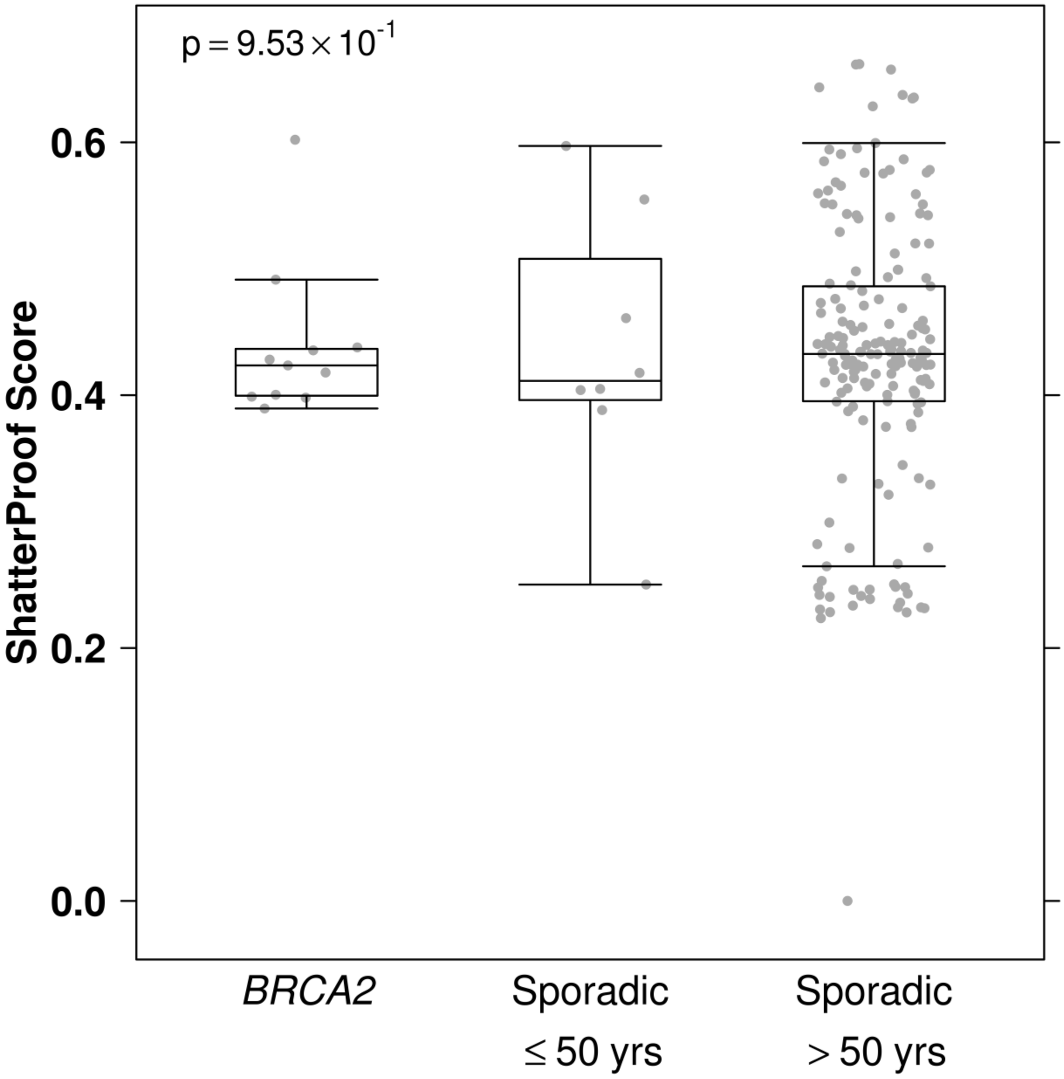

Supplementary Figure 8 | Chromothripsis rates

Boxplot comparing chromothripsis scores, generated by ShatterProof, between *BRCA2*–mutant (n = 11) and sporadic (n = 133) PCa specimens. P-value is from a two-sided, unpaired t-test. Whiskers indicate the maximum and minimum values, the box outline indicates the third and first quartile and the bar indicates the mean.

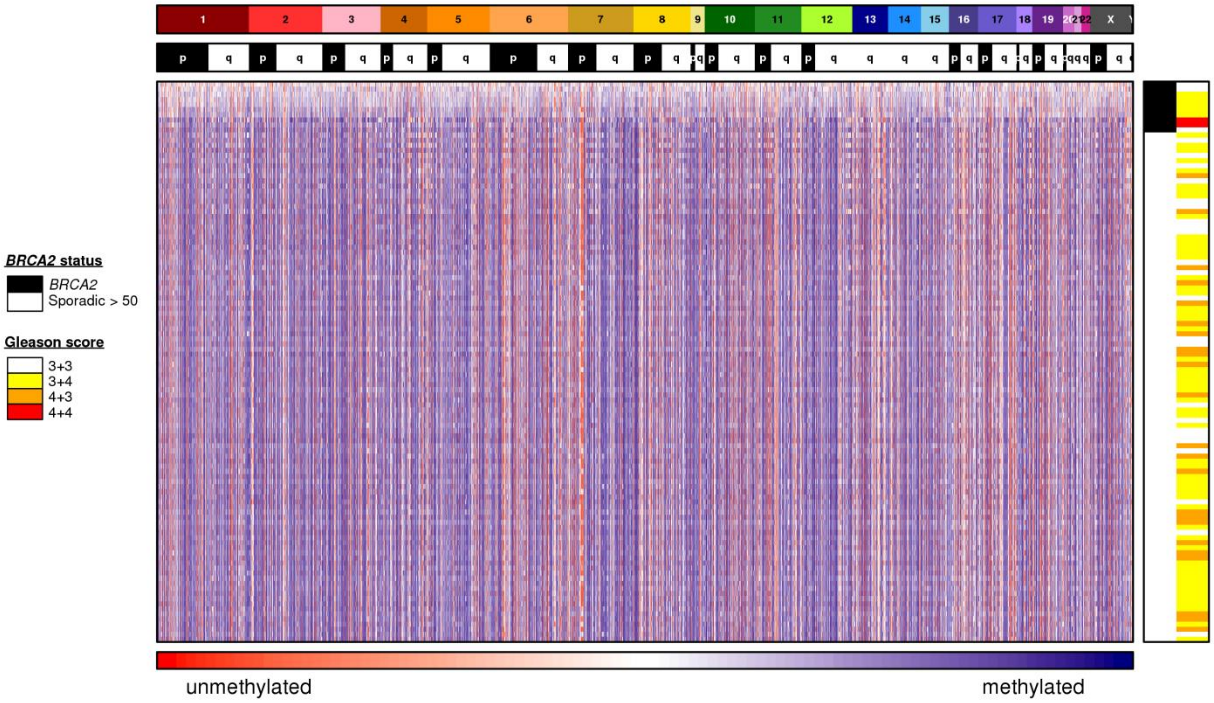

Supplementary Figure 9 | Differentially methylated probes in *BRCA2*-mutant

Heatmap of 7,445 significantly (q-value < 0.05, |log2 β-FC| > 0.1) differentially methylated probes between *BRCA2*-mutant (n = 10) and sporadic PCa (n = 100) specimens. Red represents β-values closer to 0 (unmethylated) and blue represents β-values closer to 1 (methylated). Probes (columns) were sorted by chromosome location and cytoband. Specimens (rows) were sorted based on *BRCA2* genotype and the sum of β-value in increasing order. Gleason scores are shown for each specimen.

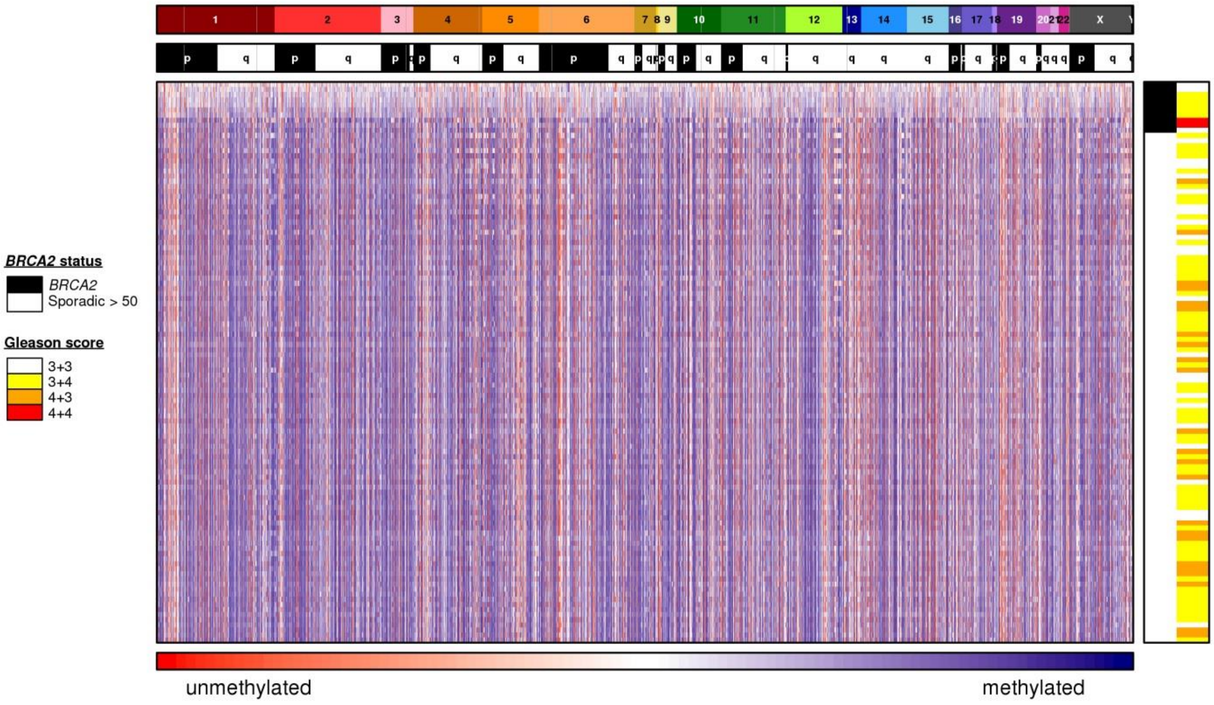

Supplementary Figure 10 | Differentially methylated probes controlling for CNA differences in *BRCA2*-mutant PCa

Heatmap of 4,979 significantly (q-value < 0.05, |log2 β-FC| > 0.1, CNA fraction < 0.1) differentially methylated probes between *BRCA2*-mutant (n = 10) and sporadic (n = 100) specimens. Red represents β-values closer to 0 (unmethylated) and blue represents β-values closer to 1 (methylated). Probes (columns) were sorted by chromosome location and cytoband. Specimens (rows) were sorted based on *BRCA2* status and the sum of β-value in increasing order. Gleason score is also shown for each specimen.

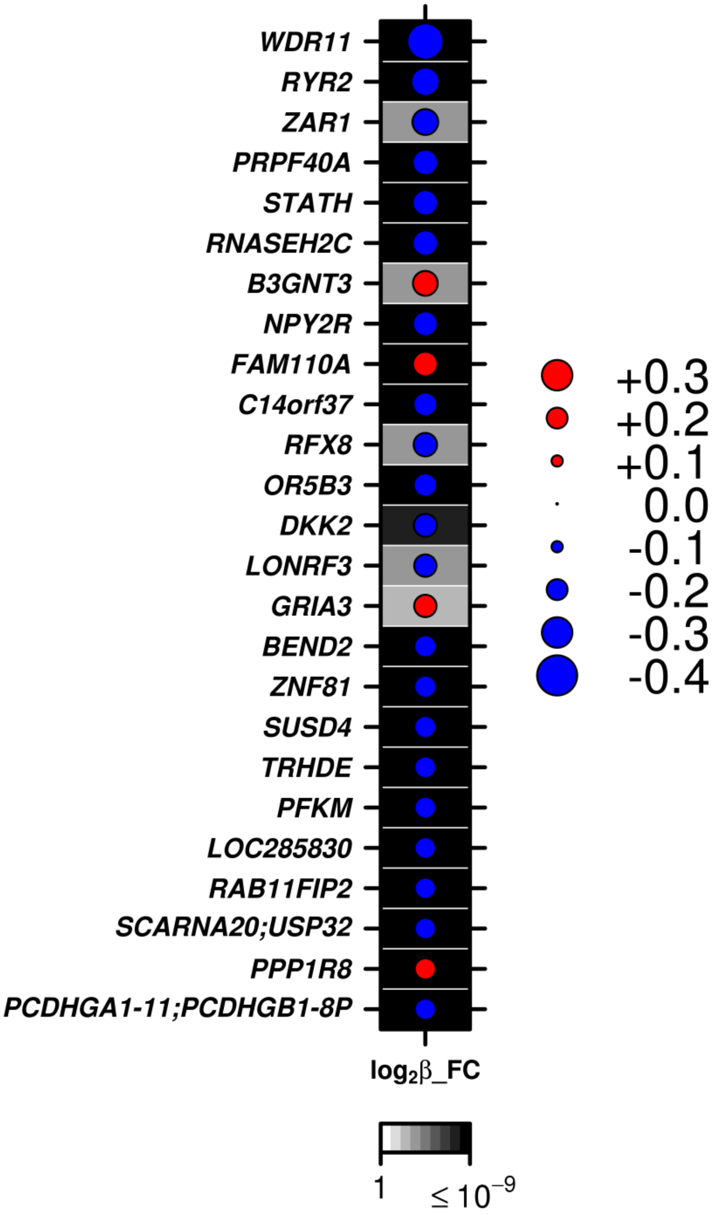

Supplementary Figure 11 | Differentially methylated genes in *BRCA2*-mutant PCa

A dotmap of the 25 most significantly differentially methylated genes between *BRCA2*-mutant and sporadic PCa, after controlling for CNA status. FDR values are represented by the shade of the gray boxes, and $\log_2$ beta fold changes are indicated by the size of the circle within each box. The genes are ordered based on $\log_2$ fold-change of the β-values.

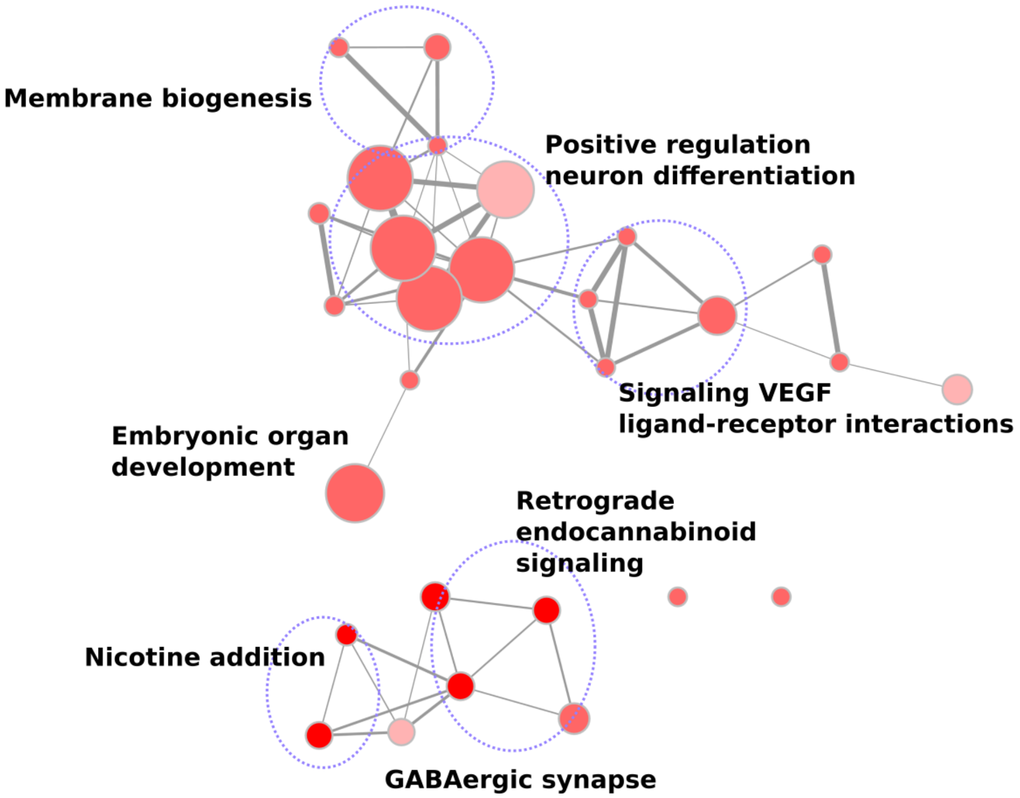

Supplementary Figure 12 | Pathway analysis of differential methylation

Illustration of the enriched pathways ($p < 0.05$) from g:Profiler analysis. Pathways were curated using gene ontology: biological process, KEGG and REACTOME. The outputs were visualized using the enrichment map in Cytoscape. Each node represents a gene-set, which is defined as a set of genes underlying a functional profile. Node size indicates the number of genes in the gene-set. Node colour represents significance of enrichment (hypergeometric test) from $p = 8.25 \times 10^{-5}$ to $p = 0.05$ (red to pink). Gene-sets are connected by a grey line if they share common genes, and line thickness reflects the number of overlapping genes. Gene-sets with similar functions are grouped together by purple dotted circles.

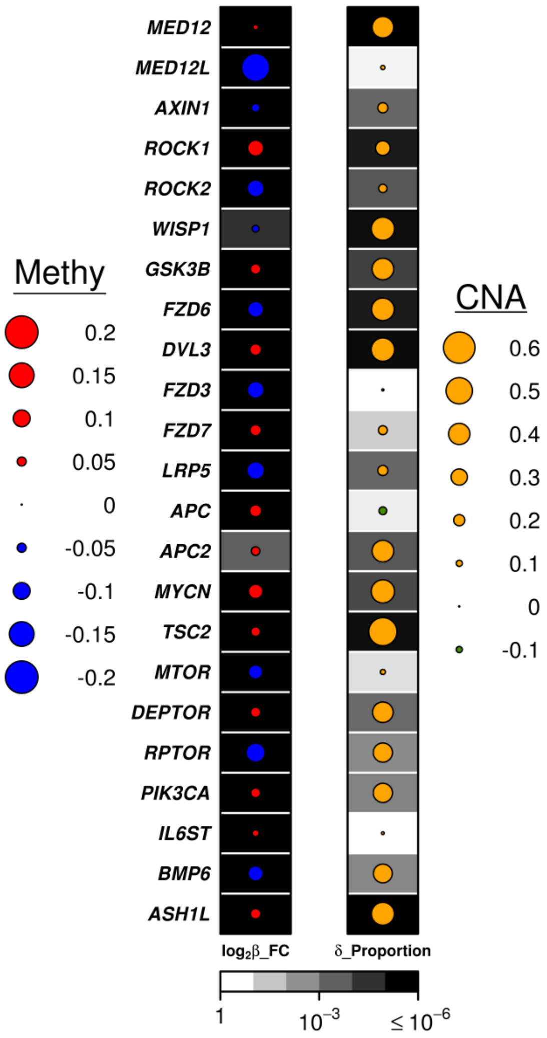

Supplementary Figure 13 | Aberrant methylation and CNAs in NED associated pathways

Key neuroendocrine differentiation (NED) associated genes show either differential methylation or a different proportion of patients with a CNA in *BRCA2*–mutant PCa relative to sporadic PCa. The shading of the grey boxes represents q-values, ranging from 0 to 1. The size of the circle for methylation analysis (left) indicates $\log_2$ beta fold change and for CNA analysis (right) it represents the difference in the proportion of patients with CNA in *BRCA2*-mutant *vs.* sporadic PCa.

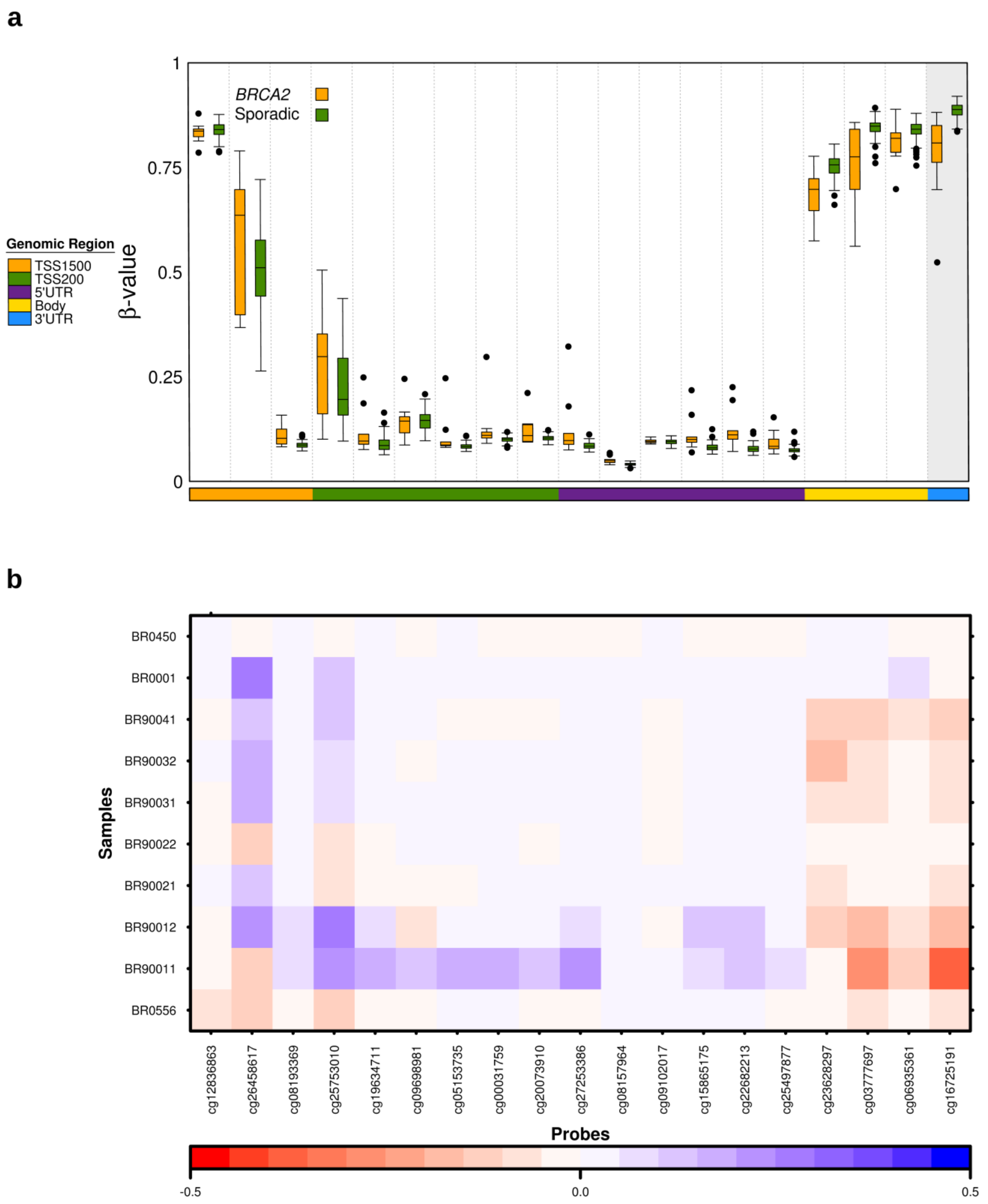

Supplementary Figure 14 | Comparative methylation of the *BRCA2* locus

(a) β-values (y-axis) of probes within the *BRCA2* locus for patients with *BRCA2*-mutant (n=10) and sporadic PCa (n = 100). Probes are sorted by genomic region. Those in grey were significantly differentially methylated ($q < 0.05$, $|\log_2 \text{FoldChange}| > 0.1$). **(b)** Heatmap of relative methylation β-values for *BRCA2*-mutant *vs.* the mean of sporadic PCa for probes shown in **(a)**.

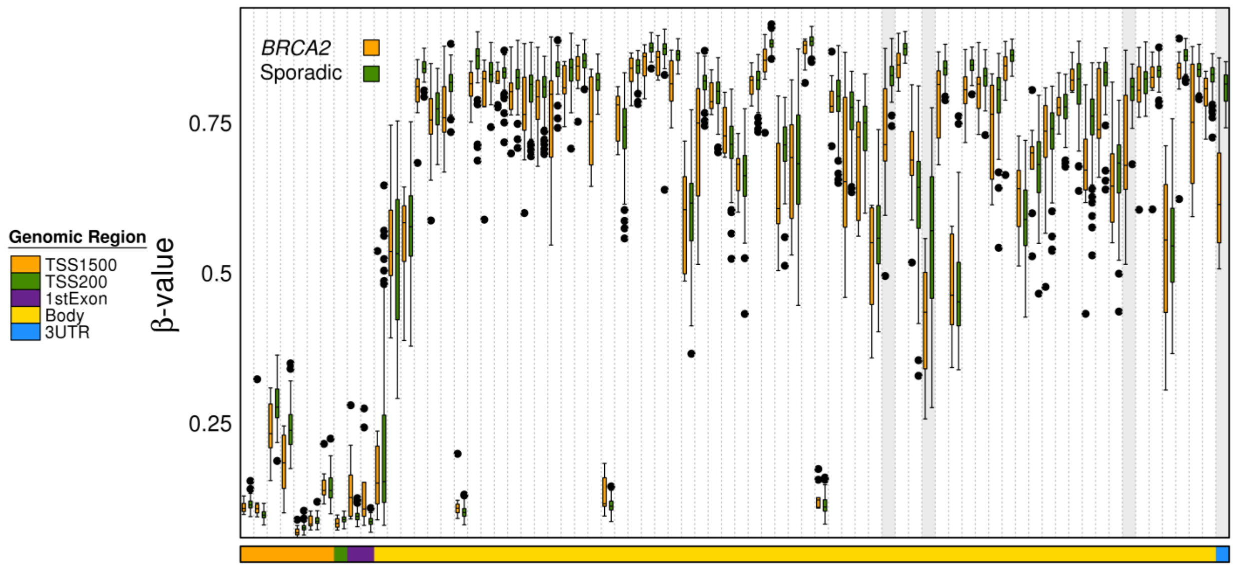

Supplementary Figure 15 | Aberrant methylation in *MED12L*

MED12L is hypomethylated in *BRCA2*-mutant PCa. β-values (y-axis) of patients with (n=10) and without *BRCA2*-mutations (n = 100) are shown. Probes highlighted in grey were shown to be significantly differentially methylated ($q < 0.05$, $|\log_2 \beta\text{-FC}| > 0.1$). Probes were sorted based on genomic region (TSS1500, TSS200, 1st Exon, gene body and 3' UTR).

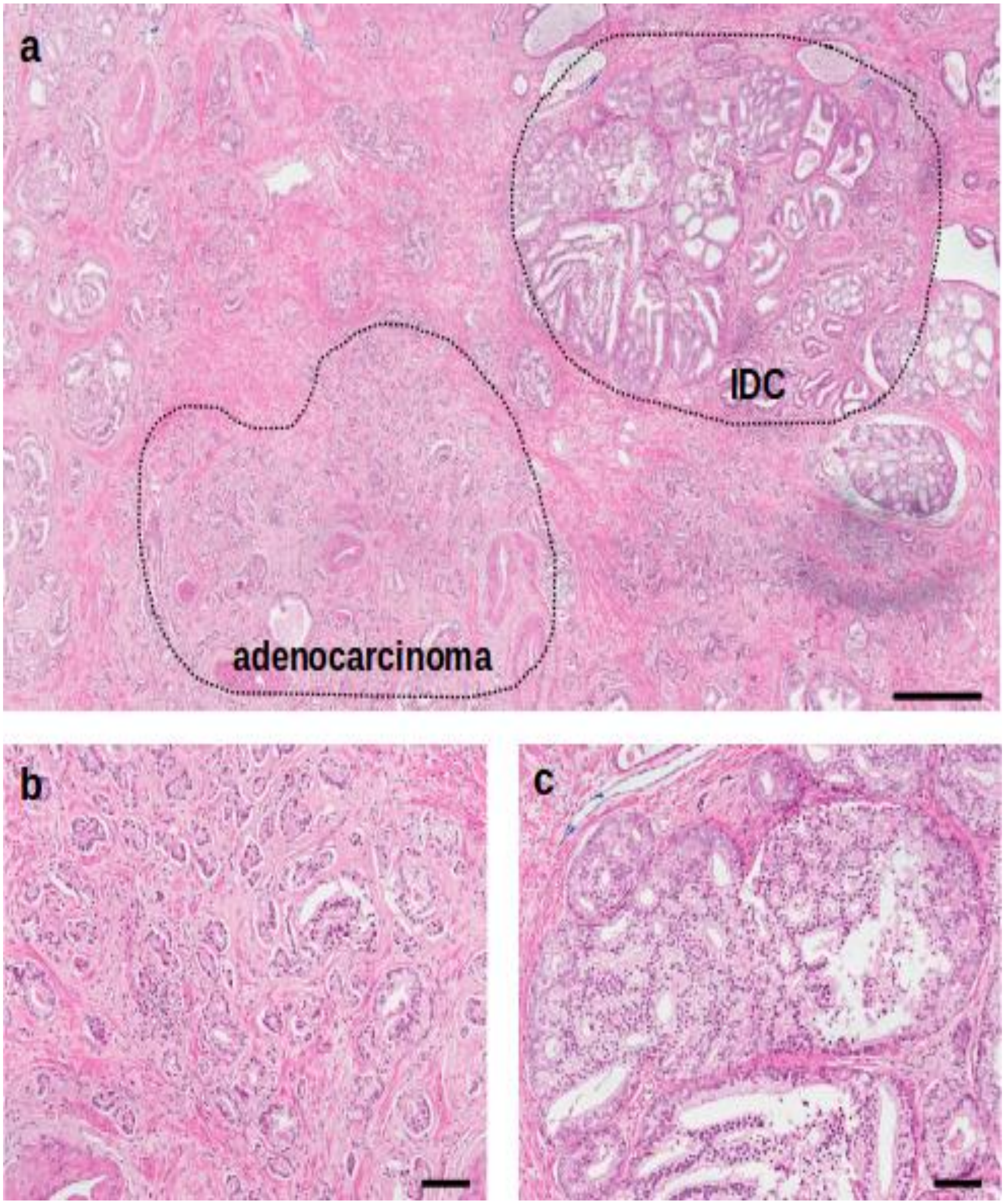

Supplementary Figure 16 | Histology of intraductal carcinoma of the prostate

(**a**) Low magnification image of haematoxylin and eosin stained tissue from patient BR9003 showing prominent areas of adenocarcinoma and IDC. (**b,c**) Higher magnification images of the regions of **(b)** Gleason 3+4 adenocarcinoma and **(c)** IDC. Individual areas such as **(b)** and **(c)** were marked by a pathologist and micro-dissected from FFPE tissues for collection of DNA.
Scale bar **(a)** 500 µm and **(b, c)** 100 µm.

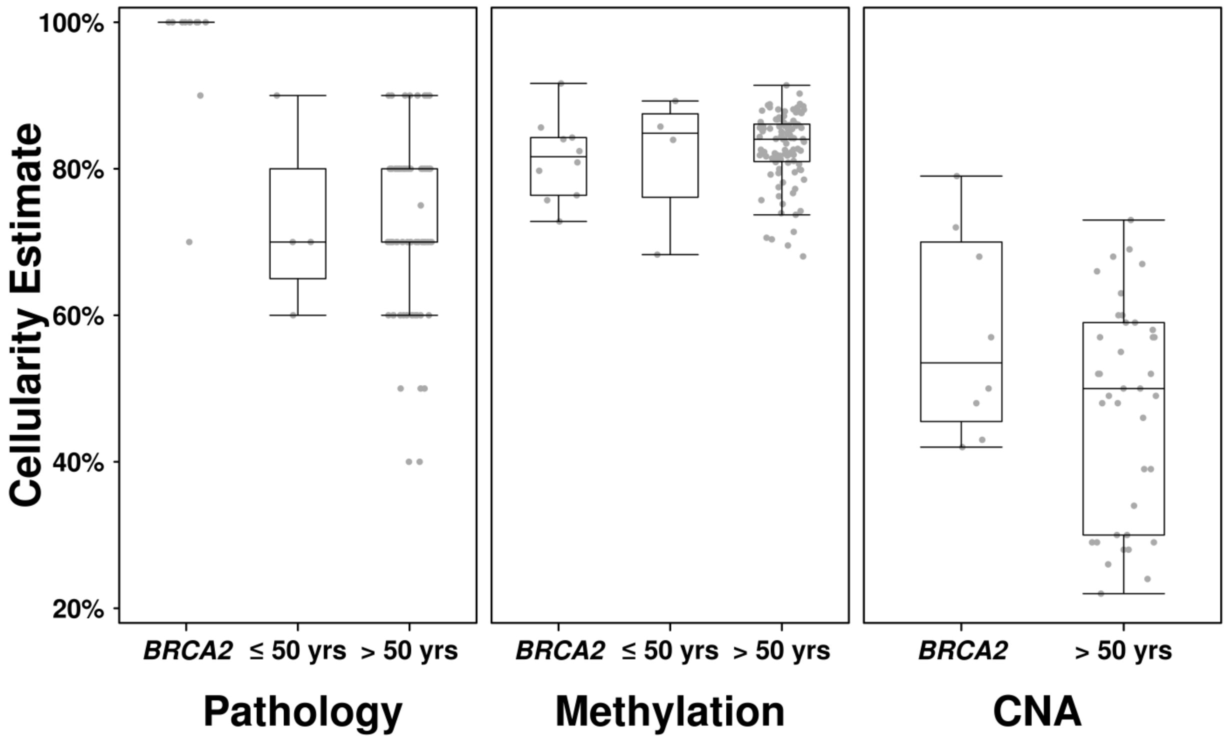

Supplementary Figure 17 | Cellularity comparisons

Cellularity estimates in specimens from *BRCA2*-mutant and sporadic PCa derived from pathology, methylation and CNAs. Sporadic specimens were stratified by age at initial treatment. Whiskers indicate the maximum and minimum values, the box outline indicates the third and first quartile and the bar indicates the mean.

Patient ID	Tumour/Germline	Tissue Type	Coverage	Lanes Sequenced	Aligned Reads
**BR9001**	Tumour	Invasive Carcinoma	39.74X	5	2381648692
	Tumour	Intraductal Carcinoma	46.24X	4	1863118468
	Germline	Benign Prostate Epithelium	41.15X	6	2663539137
**BR9002**	Tumour	Intraductal Carcinoma	51.13X	4	1764713863
	Germline	Whole Blood	47.23X	4	1647630156
	Tumour	Invasive Carcinoma	42.43X	4	1742662384
**BR9003**	Tumour	Intraductal Carcinoma	41.56X	4	1963330346
	Germline	Whole Blood	49.62X	4	1738170310
	Tumour	Invasive Carcinoma	51.60X	4	1808253685
**BR9004**	Tumour	Invasive Carcinoma	41.42X	4	1832673119
	Tumour	Intraductal Carcinoma	45.11X	4	1824591030
**BR9005**	Tumour	Invasive Carcinoma	45.44X	4	1706563103
	Tumour	Intraductal Carcinoma	36.09X	4	1476458935
	Germline	Whole Blood	48.80X	4	1754897689
**BR9013**	Tumour	Gross Tumour			
**BR9014**	Tumour	Gross Tumour			
**BR9015**	Tumour	Gross Tumour			
**BR9016**	Tumour	Gross Tumour			
**BR9017**	Tumour	Gross Tumour			
**BR9018**	Tumour	Gross Tumour			
**BR0001**	Tumour	Gross Tumour			
**BR0450**	Tumour	Invasive Carcinoma	60.42X	7	2276150296
	Germline	Whole Blood	41.42X	4	1344879117
**BR0556**	Tumour	Invasive Carcinoma	21.37X	3	728101594
**SP0100**	Tumour	Intraductal Carcinoma	29.75X	8	1396659680
	Tumour	Invasive Carcinoma	39.44X	9	1693161504
	Germline	Whole Blood	48.93X	4	1606069684
**SP0196**	Tumour	Intraductal Carcinoma	69.09X	6	2417472778

	Tumour	Invasive Carcinoma	62.24X	6	2037313683
	Germline	Whole Blood	42.28X	4	1435208636
**SP0260**	Tumour	Intraductal Carcinoma	41.45X	6	2081634607
	Tumour	Invasive Carcinoma	48.47X	6	2377641079
	Germline	Whole Blood	36.96X	4	1270011652
**SP0334**	Tumour	Intraductal Carcinoma	38.13X	6	2032242648
	Tumour	Invasive Carcinoma	44.16X	6	2210749123
	Germline	Whole Blood	35.06X	3	1135742587
**SP0361**	Tumour	Intraductal Carcinoma	59.90X	6	2453981151
	Tumour	Invasive Carcinoma	39.37X	6	2644949400
	Germline	Whole Blood	45.49X	3	1474565417
**SP0364**	Tumour	Intraductal Carcinoma	41.07X	6	1784739292
	Tumour	Invasive Carcinoma	33.97X	6	1876753050
	Germline	Whole Blood	44.65X	3	1438418976

**Patient ID**	**# Somatic SNVs UTR**	**# Somatic Non-synonymous SNVs**	**# Somatic Splicing SNVs**	**# Somatic Stopgain-loss SNVs**	**# Somatic Synonymous SNVs**	**Total Somatic SNVs (Post Filter)**
**BR9001**	26	28	0	2	7	3946
	25	26	0	2	6	4081
**BR9002**	0	0	0	0	1	129
	12	10	0	1	1	1677
**BR9003**	12	10	0	1	6	1562
	16	12	0	1	5	1921
**BR9004**						
**BR9005**	23	17	1	2	0	2654
	22	16	0	3	2	2751
**BR9013**						
**BR9014**						
**BR9015**						

**BR9016**						
**BR9017**						
**BR9018**						
**BR0001**						
**BR0450**	2	5	0	0	5	679
**BR0556**						
**SP0100**	27<br>37	15<br>17	0<br>0	0<br>0	7<br>8	3636<br>4984
**SP0196**	17<br>12	19<br>11	2<br>1	2<br>1	6<br>5	2463<br>1718
**SP0260**	9<br>7	16<br>17	1<br>1	1<br>1	7<br>8	2152<br>1837
**SP0334**	13<br>11	12<br>10	0<br>0	0<br>1	4<br>4	1817<br>1716
**SP0361**	20<br>12	13<br>12	0<br>0	0<br>2	3<br>10	1669<br>1317
**SP0364**	20<br>17	18<br>21	0<br>0	2<br>2	12<br>9	2818<br>2684

**Patient ID**	**Deletion**	**Inversions**	**Duplication**	**Translocation**	**Total GRs**	**Shatter-Proof Score**
**BR9001**	1<br>1	1<br>4	0<br>0	5<br>3	7<br>8	0.43779<br>0.39884
**BR9002**	2<br>8	2<br>7	1<br>2	14<br>8	19<br>25	0.40035<br>0.42354

**BR9003**	5	0	7	16	28	0.43547
	12	2	7	20	41	0.38948
**BR9004**	20	17	10	58	105	0.41792
	25	21	16	64	126	0.49139
**BR9005**	14	5	4	5	28	0.42815
	20	8	4	10	42	NA
**BR9013**						
**BR9014**						
**BR9015**						
**BR9016**						
**BR9017**						
**BR9018**						
**BR0001**						
**BR0450**	2	6	2	13	23	0.39802
**BR0556**	104	47	42	25	218	0.60204
**SP0100**	172	19	120	13	324	
	230	21	290	17	558	
**SP0196**	51	31	32	8	122	
	38	27	38	8	111	
**SP0260**	47	48	34	31	160	
	45	41	31	30	147	
**SP0334**	23	9	8	10	50	
	21	8	5	12	46	
**SP0361**	23	17	10	14	64	
	27	14	17	13	71	

**SP0364**	12<br>12	15<br>16	9<br>8	10<br>11	46<br>47	

Patient ID	T2E	Gleason Score	PSA	T Category	BRCA2 Genotype	Mutation Effect	ENIGMA Classification
**BR9001**		3+3	N/A	pT3a	c.7757G>A	Stop Gain	Class 5 - Pathogenic
**BR9002**		3+4	5.8	pT2c	c.778_779delGA	Frameshift	Class 5 - Pathogenic
**BR9003**	DEL; chr21:39835082-42874898	3+4	5	pT3a	c.5279C>G	Stop Gain	Class 5 - Pathogenic
**BR9004**	INV; chr21:39869852-42869523	4+5	3.4	pT3a	c.8585dupT	Frameshift	Class 5 - Pathogenic
**BR9005**		4+5	5.2	pT3a	c.9294C>G	Stop Gain	Class 5 - Pathogenic
**BR9013**		5+5	2	pT3a	c.5073dupA	Frameshift	Class 5 - Pathogenic
**BR9014**		5+5	0.4	T3b	c.8297delC	Frameshift	Class 5 - Pathogenic
**BR9015**		4+3	21	T2b	c.9154C>T	Missense	Class 5 - Pathogenic
**BR9016**		4+3	5.3	T3a	c.9117G>A	Frameshift	Class 5 - Pathogenic
**BR9017**		4+5	9.4	T2c	c.5946delT	Frameshift	Class 5 - Pathogenic
**BR9018**		4+3	3.4	T3c	c.3847_3848delGT	Frameshift	Class 5 - Pathogenic
**BR0001**		4+4	9.79	T1c	c.6174delT	Frameshift	Class 5 - Pathogenic

**BR0450**	DEL;chr21:39906213-42875068	3+3	3.03	T1c	c.5946delT	Frameshift	Class 5 - Pathogenic
**BR0556**		4+4	28.71	T3a	c.5946delT	Frameshift	Class 5 - Pathogenic
**SP0100**		4+5	5.56	T2a			
**SP0196**		4+3	4.88	T2b			
**SP0260**		4+3	6.59	T2a			
**SP0334**		3+4	7.41	T2a			
**SP0361**		3+4	4.6	T2a			
**SP0364**		3+3	8.11	T2b			

Patient ID	Number of Tumour Specimens	IDC-P?	Germline DNA Available?	Methylation Data Available ?	BRCA2 Promoter Methylation Status
**BR9001**	2	Yes	Yes	Yes<br>Yes<br>No	Hyper<br>Hyper<br>N/A
**BR9002**	2	Yes	Yes	Yes<br>No<br>Yes	Hypo<br>N/A<br>Hyper
**BR9003**	2	Yes	Yes	Yes<br>No<br>Yes	Hyper<br>N/A<br>Hyper
**BR9004**	2	Yes	No	Yes<br>No	Hyper<br>N/A
**BR9005**	2	Yes	Yes	No<br>No	N/A<br>N/A

				No	N/A
**BR9013**	1	Yes	Yes	No	N/A
**BR9014**	1	Yes	No	No	N/A
**BR9015**	1	No	Yes	No	N/A
**BR9016**	1	No	Yes	No	N/A
**BR9017**	1	Yes	Yes	No	N/A
**BR9018**	1	No	Yes	No	N/A
**BR0001**	1	No	No	Yes	Hyper
**BR0450**	1	No	Yes	Yes<br>No	Hypo<br>N/A
**BR0556**	1	No	No	Yes	Hypo
**SP0100**	2	Yes	Yes	No<br>No<br>No	N/A
**SP0196**	2	Yes	Yes	No<br>No<br>No	N/A
**SP0260**	2	Yes	Yes	No<br>No<br>No	N/A
**SP0334**	2	Yes	Yes	No<br>No<br>No	N/A
**SP0361**	2	Yes	Yes	No<br>No<br>No	N/A
**SP0364**	2	Yes	Yes	No<br>No<br>No	N/A

Patient ID	# of 1st Degree rels with verified Ca/type	# of 2nd Degree rels with verified Ca/type
**BR9001**	n=9: x5 BrCa, x1 BrCa/Oesph, x2 PC, x1 Bowel	n=1: x1 Ov/BrCa
**BR9002**	n=1: x1 Brca	n=5: x2 Brca, x1 PC, x1 unknown primary, x1 lung
**BR9003**	n=4: x3 BrCa, x1 PC	n=1: x1 OvCa
**BR9004**	n=2: x1 BrCa, x1 BrCa/OvCa	n=0
**BR9005**	n=2: x2 BrCa	n=2; x2 BrCa
**BR9013**	n=2: x2 BrCa	n=7: x1 Rectal/BrCa, x2 PC, x1 OvCa, x3 BrCa
**BR9014**	n=1: x1 unknown primary	n=2: x1 BrCa, x1 bowel
**BR9015**	n=1: x1 PC	n=4: x1 Br/OvCa, x3 PancCa,
**BR9016**	n=2: x1 PC, x1 Bowel	n=2: x1 BrCa, x1 Lung
**BR9017**	n=3: x1 PC, x1 Thyroid & BrCa, x1 Uterine	n=1: x1 bowel
**BR9018**	n=2: x1 PC, x1 unknown primary	n=5: x2 BrCa, x1 OvCa, x1 Pancreatic, x1 Bowel
**BR0001**	Unknown	Unknown
**BR0450**	Unknown	Unknown
**BR0556**	Unknown	Unknown
**SP0100**	N/A	N/A

**SP0196**	N/A	N/A
**SP0260**	N/A	N/A
**SP0334**	N/A	N/A
**SP0361**	N/A	N/A
**SP0364**	N/A	N/A

Supplementary Table 1 | Summary and clinical data

Summary data on sequencing statistics for the 19 specimens from the 14 *BRCA2*-mutant patients and 12 specimens from six sporadic patients including tissue type, coverage, number of lanes sequenced, and total aligned reads. The number of somatic SNVs in coding (nonsynonymous, synonymous, and stop-gain), splicing, and UTR regions predicted and annotated using SomaticSniper and Annovar, respectively. Also included is the number of various types of somatic genomic rearrangements including deletions, inversions, duplications, and interchromosomal translocations called using Delly. Chromothripsis scores were calculated using ShatterProof, while the presence of TMPRSS2-ERG fusions was determined by examining breakpoints on chromosome 21:39-42 Mbp. Clinical data is also provided, including the exact *BRCA2* mutation position and mutational effect, Gleason score, pre-treatment PSA level, pre-treatment T stage, and histological diagnosis. Availability of germline DNA and number of DNA specimens are also indicated.

	***BRCA2*-mutant vs. sporadic (< 50 yrs)**		***BRCA2*-mutant vs. sporadic (> 50 yrs)**	
	**P-value**	**Effect Size**	**P-value**	**Effect Size**
CNA count	0.1	17.5	0.004	25.5
GR count	0.000845809	25	0.05085553	8
SNV count	0.01156451	1079.5	0.04023714	392
PGA	0.000389816	15.92	2.80E-006	13.5

Supplementary Table 2 | Statistical analyses of mutational burden.

Wilcoxon rank-sum test p-values comparing measures of mutational burden (including number of CNAs, GRs, SNVs, and PGA) between *BRCA2*-mutant and sporadic PCa.

ID	Description	T (number of term genes)	Q (number of guery genes)	Q&T (number of common genes)	Total gene list	Enrichment	FDR	Q&T list
KEGG: 05032	Morphine addiction	91	2601	33	20755	2.894	< 0.001	GNAS,GNG4,GNG12,PDE1A,PDE4B,PDE2A,GABRE,PDE3B,GNB2,PDE8A,GNGT1,GNB5,ADCY9,PRKCB,PRKX,GABRA4,GNAI3,GABRA5,ADCY6,ADRBK1,CACNA1A,PDE11A,GABRG3,GABRG2,GABRB3,KCNJ3,PDE10A,PRKCG,PDE7B,SLC32A1,OPRM1,PDE1B,KCNJ5
KEGG: 04723	Retrograde endocannabinoid signaling	101	2601	32	20755	2.528	< 0.001	GRIA3,GRIA4,GNG4,GNG12,GABRE,GNB2,GRM1,GNGT1,GNB5,ADCY9,PRKCB,PRKX,GABRA4,GNAI3,GABRA5,ADCY6,CACNA1A,ITPR1,MAPK10,GABRG3,GRIA1,GABRG2,GABRB3,KCNJ3,MAPK8,PRKCG,SLC32A1,PTGS2,GRIA2,GNAQ,SLC17A8,KCNJ5
KEGG: 04713	Circadian entrainment	96	2601	30	20755	2.494	< 0.001	RYR2,GRIA3,GRIA4,GNAS,GNG4,GNG12,PRKG1,GNB2,GNGT1,GNB5,ADCY9,PRKCB,PRKX,GNAI3,ADCY6,ITPR1,PER3,RPS6KA5,CAMK2B,GRIA1,NOS1AP,KCNJ3,PRKCG,GRIN2C,GRIA2,GRIN2A,RYR3,GNAQ,RYR1,KCNJ5
KEGG: 04724	Glutamatergic synapse	114	2601	32	20755	2.240	< 0.001	GRIA3,GRM6,GRIA4,GNAS,GNG4,GNG12,GNB2,GRM1,GNGT1,GNB5,P

								PP3CA,ADCY9,PRKCB,SHANK2,PRKX,GNAI3,SLC1A3,GRIK3,ADCY6,DLGAP1,ADRBK1,CACNA1A,ITPR1,GRIA1,KCNJ3,PRKCG,GRIN2C,GRIA2,GRIN2A,GNAQ,SLC1A2,SLC17A8
KEGG: 05033	Nicotine addiction	39	2601	15	20755	3.069	0.002	GRIA3,GRIA4,GABRE,GABRA4,GABRA5,CACNA1A,GABRG3,GRIA1,GABRG2,GABRB3,GRIN2C,SLC32A1,GRIA2,GRIN2A,SLC17A8
GO:0051094	positive regulation of developmental process	470	2601	94	20755	1.596	0.005	NRXN1,WNT7A,TGFBR2,SEMA5A,HMGB2,ANXA3,RPS6KA3,ODZ4,GHRL,IFNG,SOX11,DMD,GATA3,FBXW8,NEUROD1,TNFSF4,HIPK1,SRY,PRKCB,PROM1,GCNT2,BMP10,TWIST1,EPHB2,TFAP2A,SMYD1,PAX8,BASP1,POU4F2,RUNX1,DDR2,DKK1,NUMB,NELL1,CASP8,VHL,GLI2,ZEB1,INSM1,BMP4,MYOG,CTNNA1,ETS1,BDNF,NKX6-1,BHLHB9,GREM1,SOX6,NRP1,THBS1,MKL2,HMGA2,SOX9,OTX2,MAP2K6,ASB4,BCL9L,F3,TBX5,IL1RAPL1,ARNTL,FLT1,CD53,HDAC9,CAMK2B,PTPRD,NKX2-5,VNN1,ATOH1,MUL1,ZBTB1,TAL1,VWC2,ANGPT4,HOXD3,MFF,RBPJ,CTGF,COL1A1,NPTN,ASXL2,ACVR1,PTGS2,RGS14,BMP6,LRP5,TMEM100,NGF,TCF7L2,BMP5,ACVR2A,PACS

								IN1,OPRM1,BMP2
GO:0007416	synapse assembly	56	2601	20	20755	2.850	0.006	NRXN1,WNT7A,GHRL,SHANK2,NLGN4X,EPHB2,PCDHB3,BDNF,BHLHB9,DNM3,IL1RAPL1,PTPRD,PCDHB13,NRXN2,PCDHB5,PDLIM5,LRP4,PCDHB14,NLGN4Y,PCDHB2
GO:0060284	regulation of cell development	297	2601	65	20755	1.746	0.006	ID2,WNT7A,SEMA5A,ODZ4,IFNG,SOX11,DMD,FBXW8,NEUROD1,SARM1,FGF13,GCNT2,BMP10,RAPGEF1,KIAA0319,EPHA4,TWIST1,CNOT2,SMYD1,CHN1,PAX8,CDK1,NUMB,ZEB1,BMP4,MYOG,LTK,GFI1,PAX6,NEDD4,NKX6-1,SIPA1L1,BHLHB9,GREM1,NRP1,CNTN4,COL3A1,LRRC4C,HMGA2,SOX9,BCL9L,TBX5,ELL3,IL1RAPL1,ARNTL,HDAC9,CAMK2B,PTPRD,NKX2-5,ATOH1,SOX3,TRPV4,VWC2,RUFY3,PDLIM5,HOXD3,CCDC88A,COL1A1,NPTN,RGS14,LRP4,NGF,SSH2,TCF7L2,KLK8
REAC:74736	GRB2:SOS binds IRS-P	4	2590	4	20755	8.014	0.008	IRS1,IRS2,SOS1,GRB2
GO:0002067	glandular epithelial cell differentiation	18	2601	10	20755	4.433	0.009	NEUROD1,CDK6,INSM1,BMP4,PAX6,NKX6-1,ARNTL,PDX1,BMP6,BMP5
GO:0050767	regulation of neurogenesis	214	2601	50	20755	1.864	0.010	ID2,WNT7A,SEMA5A,ODZ4,SOX11,DMD,FBXW8,NEUROD1,SARM1,FGF13,RAPGEF1,KIAA0319,EPHA4,CHN1,CDK1,NUMB,ZEB1,LTK,GFI1,PAX6,N

								EDD4,SIPA1L1,BHLHB9,NRP1,CNTN4,COL3A1,LRRC4C,SOX9,IL1RAPL1,ARNTL,CAMK2B,PTPRD,NKX2-5,ATOH1,SOX3,TRPV4,VWC2,RUFY3,PDLIM5,HOXD3,CCDC88A,NPTN,RGS14,LRP4,NGF,SSH2,KLK8,BMP5,PACSIN1,OPRM1
GO:0071709	membrane assembly	12	2601	8	20755	5.320	0.010	NRXN1,ANK3,NLGN4X,IL1RAPL1,PTPRD,NRXN2,SPTBN1,LRP4
GO:0035883	enteroendocrine cell differentiation	15	2601	9	20755	4.788	0.011	NEUROD1,CDK6,INSM1,BMP4,PAX6,NKX6-1,ARNTL,PDX1,BMP6
GO:0044091	membrane biogenesis	15	2601	9	20755	4.788	0.011	NRXN1,ANK3,NLGN4X,IL1RAPL1,PTPRD,NRXN2,SPTBN1,LRP4,CLIP3
GO:2000679	positive regulation of transcription regulatory region DNA binding	7	2601	6	20755	6.840	0.012	GATA3,NEUROD1,HAND2,TWIST1,HMGA2,TGFB1
GO:0051960	regulation of nervous system development	239	2601	54	20755	1.803	0.013	ID2,NRXN1,WNT7A,SEMA5A,ODZ4,GHRL,SOX11,DMD,FBXW8,NEUROD1,SARM1,FGF13,RAPGEF1,KIAA0319,EPHA4,EPHB2,CHN1,CDK1,NUMB,ZEB1,LTK,GFI1,PAX6,NEDD4,BDNF,SIPA1L1,BHLHB9,NRP1,CNTN4,COL3A1,LRRC4C,SOX9,IL1RAPL1,ARNTL,CAMK2B,PTPRD,NKX2-5,ATOH1,SOX3,TRPV4,VWC2,RUFY3,PDLIM5,HOXD3,CCDC88A,NPTN,RGS14,LRP4,NGF,SSH2,KLK8,BMP5,PACSIN1,OPRM

								1
KEGG: 04728	Dopaminergic synapse	130	2601	31	20755	1.903	0.018	GRIA3,GRIA4,GNAS,GNG4,GNG12,PPP2R2B,GNB2,GNGT1,GNB5,PPP3CA,PRKCB,PRKX,GNAL,GNAI3,CACNA1A,ITPR1,KIF5C,MAPK10,PPP2R5C,ARNTL,SLC18A2,ATF2,CAMK2B,GRIA1,KCNJ3,MAPK8,PRKCG,GRIA2,GRIN2A,GNAQ,KCNJ5
GO:0048568	embryonic organ development	122	2601	32	20755	2.093	0.028	RYR2,ID2,TGFBR2,STIL,TCF21,SOX11,NEUROD1,HAND2,DSCAML1,TWIST1,EPHB2,TFAP2A,PAX8,KDM2B,CASP8,GLI2,SOX17,SOX9,RARB,COL2A1,KIT,TGFBR1,PKD2,NKX2-5,VANGL2,TAL1,KITLG,HOXD3,RBPJ,NIPBL,ACVR1,FUZ
REAC:194138	Signaling by VEGF	11	2590	6	20755	4.371	0.034	VEGFC,NRP1,FLT1,NRP2,FIGF,KDR
REAC:194313	VEGF ligand-receptor interactions	11	2590	6	20755	4.371	0.034	VEGFC,NRP1,FLT1,NRP2,FIGF,KDR
REAC:194306	Neurophilin interactions with VEGF and VEGFR	5	2590	4	20755	6.411	0.037	NRP1,FLT1,NRP2,KDR
REAC:109823	SOS phosphorylation and dissociation (IRS)	5	2590	4	20755	6.411	0.037	IRS1,IRS2,SOS1,GRB2
REAC:173512	I-SMAD competes with R-SMAD for type I receptor	5	2590	4	20755	6.411	0.037	TGFBR2,SMAD6,TGFBR1,TGFB1
REAC:265177	Exocyst complex formation	8	2590	5	20755	5.008	0.042	EXOC7,EXOC4,EXOC2,EXOC5,EXOC1
KEGG: 04510	Focal adhesion	208	2601	43	20755	1.650	0.043	TNXB,ACTN2,CAPN2,PIK3R3,PAK7,PRKCB,PPP1R12A,IGF1R,MYLK4,R

								APGEF1,TNR,BIRC2,COL11A2,SOS1,GRB2,VEGFC,ACTN1,PXN,SHC3,ITGA1,FN1,THBS1,MAPK10,COL3A1,COL2A1,ITGA2,FLT1,SHC1,FIGF,COL4A5,MAPK8,ITGB7,PRKCG,TLN2,PARVB,COL1A1,COL4A1,ITGA4,PDGFD,PPP1R12B,IBSP,COL11A1,KDR
KEGG: 04727	GABAergic synapse	87	2601	22	20755	2.018	0.050	GNG4,GNG12,SLC6A1,GABRE,GNB2,GNGT1,GNB5,GPHN,ADCY9,PRKCB,PRKX,GABRA4,GNAI3,GABRA5,ADCY6,CACNA1A,GABRG3,SLC6A13,GABRG2,GABRB3,PRKCG,SLC32A1
GO:0045664	regulation of neuron differentiation	181	2601	42	20755	1.852	0.050	ID2,SEMA5A,SOX11,DMD,FBXW8,NEUROD1,SARM1,FGF13,RAPGEF1,KIAA0319,EPHA4,CHN1,ZEB1,LTK,GFI1,NEDD4,SIPA1L1,BHLHB9,NRP1,CNTN4,LRRC4C,SOX9,IL1RAPL1,CAMK2B,PTPRD,NKX2-5,ATOH1,SOX3,TRPV4,VWC2,RUFY3,PDLIM5,HOXD3,CCDC88A,NPTN,LRP4,NGF,SSH2,KLK8,BMP5,PACSIN1,BMP2
REAC:75178	Formation of Platelet plug	120	2590	27	20755	1.803	0.050	GNG4,GNG12,ACTN2,GNB2,GNGT1,GNB5,PRKCB,APBB1IP,SELP,ALB,SOS1,GRB2,VEGFC,ACTN1,FGA,ITPR1,FN1,THBS1,ITGA2,LAMP2,FIGF,PPBP,COL1A1,FGG,GNAQ,GP6,TGFB1

Supplementary Table 3 | g:Profiler analysis of differentially methylated genes

Enriched pathways ($p < 0.05$) from a g:Profiler analysis including gene ontology: biological process, KEGG, and REACTOME are specified. These pathways are enriched in genes showing significant differential methylation between *BRCA2*-mutant and sporadic PCa.
