# Supplementary material for: Germline BRCA2 mutations drive prostate cancers with distinct evolutionary trajectories
Source: Nat Commun. 2017 Jan 9;8:13671. doi: 10.1038/ncomms13671 (PMC5227331; doi:10.1038/ncomms13671)
Supplement: Peer Review File [file ncomms13671-s9.pdf]

Editorial Note: this manuscript has been previously reviewed at another journal that is not operating a transparent peer review scheme. This document only contains reviewer comments and rebuttal letters for versions considered at Nature Communications.

REVIEWERS' COMMENTS:

Reviewer #3 (Remarks to the Author):

The authors have nicely incorporated previous comments. I would suggest to highlight promoter methylation in cases with BRCA2 mutations as a second hit for inactivation of this tumor suppressor gene inactivation.

This manuscript should be accepted for publication.
